# Disruption of the SNRPF–DDX24–E2F4 Feedback Loop Uncouples Splicing and Transcriptional Regulation to Suppress Ovarian Cancer Progression

**DOI:** 10.1002/advs.202523374

**Published:** 2026-05-10

**Authors:** Yingwei Li, Zhongshao Chen, Qianqian Gao, Yuehan Gao, Ning Yang

**Affiliations:** ^1^ Department of Obstetrics and Gynecology Shandong Key Laboratory of Reproductive Health and Birth Defects Prevention and Control Qilu Hospital of Shandong University Medical Integration and Practice Center Cheeloo College of Medicine Shandong University Ji'nan China; ^2^ Department of Obstetrics and Gynecology Shandong Key Laboratory of Reproductive Health and Birth Defects Prevention and Control Qilu Hospital of Shandong University Ji'nan China

**Keywords:** intron retention, ovarian cancer, RNA splicing, SNRPF, therapeutic targets

## Abstract

Ovarian cancer (OC) remains a major cause of gynecologic cancer mortality, with progress in targeted therapy limited by an incomplete understanding of post‐transcriptional oncogenic drivers. Dysregulated RNA splicing—particularly intron retention (IR)—is increasingly recognized as a key driver of tumor progression. Here, integrated transcriptomic and proteomic analyses identify SNRPF, a core spliceosomal component, as a potent oncogenic driver in OC. SNRPF is highly expressed in tumor specimens, and its overexpression predicts poor patient survival. Silencing SNRPF suppresses proliferation, invasion, and xenograft growth. IR‐focused analysis reveals that SNRPF depletion induces intron 6 retention in DDX24, disrupting the Helicase_C domain and generating premature termination codons that activate nonsense‐mediated decay (NMD), thereby reducing DDX24 protein abundance and markedly impairing its oncogenic function. DDX24 depletion similarly promotes intron 2 retention in E2F4, causing NMD‐mediated downregulation. Notably, E2F4 directly binds the SNRPF promoter, forming a self‐sustaining “SNRPF–DDX24–E2F4” axis linking splicing and transcriptional regulation. Antisense oligonucleotide‐mediated inhibition of SNRPF disrupts this feedback loop, downregulates DDX24 and E2F4 via IR, and significantly impairs tumor growth in vitro, in vivo, and in patient‐derived xenografts. These findings define a splicing–transcription coupling mechanism in OC and position SNRPF as a promising therapeutic target.

## Graphical abstract

This study identifies SNRPF as a critical oncogenic driver in ovarian cancer. By regulating a self‐sustaining SNRPF–DDX24–E2F4 feedback loop through intron retention and nonsense‐mediated decay, SNRPF couples RNA splicing with transcriptional regulation to promote tumor progression. Therapeutic targeting of this axis using antisense oligonucleotides (ASOs) effectively suppresses tumor growth, offering a promising strategy for ovarian cancer treatment.

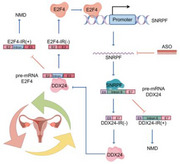

## 1 Introduction

Ovarian cancer (OC) is a major gynecological malignancy, ranking eighth worldwide in both incidence and mortality among women [1]. In the United States, projections for 2025 estimate nearly 20 890 new diagnoses and 12 730 fatalities due to OC [2]. The most predominant subtype, epithelial ovarian cancer (EOC), accounts for nearly 90% of all diagnosed cases, with high‐grade serous carcinoma (HGSOC) accounting for 70–80% of these cases. The lack of reliable early screening methods poses a significant challenge for early diagnosis, as approximately 95% of patients initially exhibit vague, nonspecific symptoms, resulting in approximately 80% of cases being diagnosed at advanced stages [1]. Standard treatment for advanced EOC involves cytoreductive surgery combined with systemic chemotherapy supplemented by personalized maintenance therapies. Targeted therapies, including antiangiogenic agents and PARP inhibitors, have emerged as pivotal components of precision medicine in OC. Nevertheless, an incomplete understanding of EOC pathogenesis limits the development of biomarker‑driven strategies. In particular, a rarely investigated yet potentially decisive subtype of alternative splicing (AS) events—intron retention (IR)—may represent a critical molecular driver of disease progression. Identifying and characterizing such unrecognized drivers could not only expand the biomarker repertoire but also provide new mechanistic insights for targeted therapeutic development aimed at improving clinical outcomes in OC.

In 99.5% of human introns, RNA splicing is catalyzed by the major (U2‐dependent) spliceosome, with the remaining 0.5% processed by the minor (U12‐dependent) spliceosome [3, 4]. AS is a pivotal post‑transcriptional mechanism that enables a single gene to yield multiple mRNA isoforms, thereby substantially expanding proteomic diversity [5, 6]. Dysregulation of AS can produce oncogenic splice variants that enhance proliferation, metastasis, and therapeutic resistance, or disrupt tumor‑suppressive isoforms, ultimately promoting malignancy. Among the diverse AS patterns, IR is characterized by the aberrant preservation of introns within mature transcripts. IR often introduces premature termination codons (PTCs), activating nonsense‑mediated decay (NMD) and consequently reducing protein abundance [7]. Alternatively, retained introns can alter protein domain architecture or generate tumor‐specific neoantigens, thereby influencing cancer biology through multiple mechanisms [8, 9, 10].

The spliceosome is a large ribonucleoprotein complex composed of small nuclear ribonucleoproteins (snRNPs), each containing seven highly conserved core Sm proteins (SNRPB, SNRPD1, SNRPD2, SNRPD3, SNRPE, SNRPF, and SNRPG) bound to small nuclear RNAs (snRNAs) [11]. Accurate splicing is essential for cellular homeostasis, and deregulation of Sm‐encoding genes is common in cancers. Several Sm proteins have been directly implicated in tumorigenesis via IR‐mediated regulation of key transcripts. For example, in non‐small cell lung cancer, SNRPB promotes tumorigenesis by modulating IR of RAB26 coupled with NMD [12]; in endometrial cancer, SNRPB influences POLD1 expression through IR, enhancing proliferative capacity [13]; and in hepatocellular carcinoma, SNRPD2 cooperates with HNRNPL to regulate IR of DDX39A, thereby sustaining elevated MYC protein levels [14].

Despite these advances, the role of Sm proteins in OC—particularly in the context of IR—remains poorly defined. To systematically identify key Sm proteins in OC, we integrated TCGA transcriptomic and CPTAC proteomic datasets, revealing robust overexpression of SNRPF in HGSOC. Although SNRPF dysregulation has been noted in other malignancies [15, 16], its functional contribution to OC, particularly via IR‐dependent mechanisms, has not been elucidated.

Given accumulating evidence that IR plays a decisive role in orchestrating oncogenic and tumor‐suppressive networks, we selected SNRPF for detailed investigation. In this study, we show that SNRPF is markedly overexpressed in OC and correlates with poor patient prognosis. Functional analyses show that SNRPF drives OC progression through dysregulated splicing–transcription coupling within a self‑reinforcing SNRPF–DDX24–E2F4 feedback loop. Moreover, Antisense oligonucleotide (ASO)‑based targeting of SNRPF effectively uncouples this oncogenic circuit, underscoring its potential as a promising therapeutic strategy.

## 2 Results

### 2.1 SNRPF is Highly Expressed in OC Tissues and Correlates With Poor Prognosis

To identify splicing factors potentially involved in OC progression, we performed differential expression analysis of OC versus normal ovary tissues using the TCGA‑OV dataset via GEPIA 3 (|log_2_FC| ≥ 1, padj < 0.05). This yielded 9,506 differentially expressed genes (DEGs), including 5232 upregulated and 4274 downregulated transcripts. Intersection of the upregulated genes with the KEGG spliceosome pathway [17] identified 18 splicing factors with elevated expression, four of which were Sm proteins (SNRPB, SNRPD1, SNRPF, SNRPG).

**FIGURE 1:**
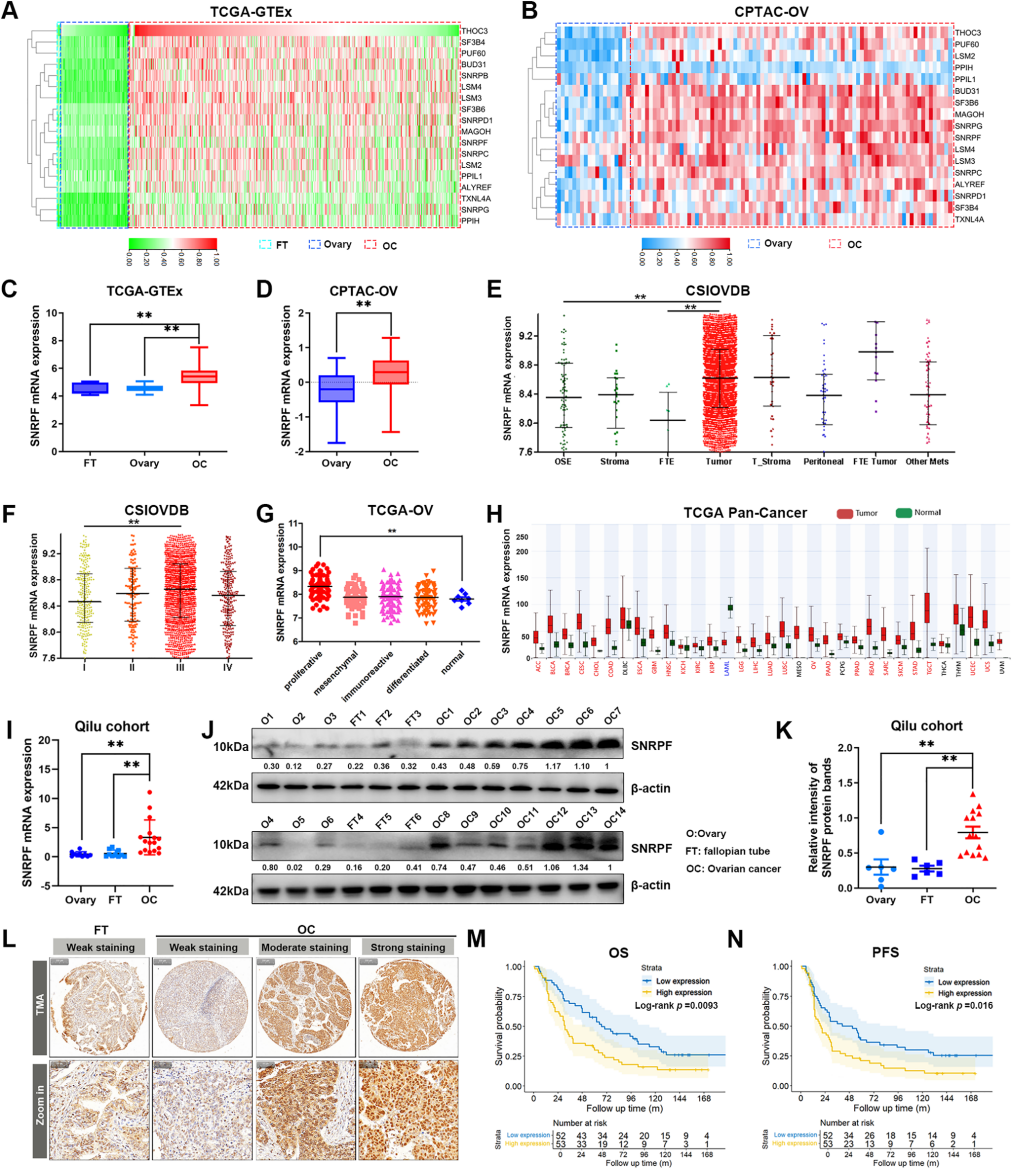
SNRPF Is Highly Expressed in OC Tissues and Is Correlated with Poor Prognosis. (A) Differential mRNA expression of 18 spliceosome‐related genes (KEGG‐selected) in normal ovary (*n* = 88, GTEx), normal FT (*n* = 5, GTEx), and OC (*n* = 426, TCGA) tissues using TCGA‐GTEx data. (B) Protein expression differences of the 18 spliceosome‐related genes between normal ovary (*n* = 19) and OC (*n* = 84) tissues based on CPTAC proteomics (data for SNRPB were unavailable). (C) Differential expression analysis of SNRPF mRNA levels in OC (*n* = 426), normal FT (*n* = 5), and ovary (*n* = 88) tissues using TCGA‐GTEx data. (D) Comparison of SNRPF protein expression levels in OC (*n* = 84) and normal ovary (*n* = 19) tissues using CPTAC proteomic data. (E) CSIOVDB (http://csiovdb.mc.ntu.edu.tw/CSIOVDB.html) analysis of SNRPF mRNA expression across various ovarian tissue types, highlighting OC vs. normal ovarian surface epithelium (OSE) and FT epithelium (FTE). (F) CSIOVDB analysis of SNRPF mRNA levels stratified by FIGO stage in OC. (G) TCGA‐OV (AffyU133a) data analysis of molecular subtype‐specific patterns of SNRPF mRNA in proliferative (*n* = 79), mesenchymal (*n* = 68), immunoreactive (*n* = 84), and differentiated (*n* = 68) subtypes, along with normal ovary (*n* = 8) tissues. (H) Pan‐cancer analysis of SNRPF mRNA expression across multiple human malignancies compared with normal tissues using the GEPIA3 database. (I) qPCR analysis of differential expression of SNRPF in normal FT (*n* = 10), normal ovary (*n* = 10), and OC tissues (*n* = 16). (J) Western blotting analysis of SNRPF protein levels in normal FT (*n* = 6), normal ovary (*n* = 6), and OC (*n* = 14) tissues. The numbers below the bands represent relative protein expression levels quantified by densitometry and normalized to β‐actin. (K) Quantification of Western blotting bands derived from panel (J). (L) Representative immunohistochemistry images showing SNRPF staining in normal FT and OC tissues. (M, N) Kaplan–Meier analysis of OS and PFS in relation to SNRPF expression, based on tissue microarray (TMA) immunostaining scores and clinical follow‐up data. Statistical significance was determined using unpaired *t*‐tests for two‐group comparisons (D, H), and one‐way ANOVA followed by Tukey's multiple comparisons test for multi‐group analyses (C, E, F, G, I, and K). Survival analysis was evaluated using the Log‐rank test (M, N). ^**^ *p* < 0.01.

Expression profiling across TCGA‑GTEx datasets revealed consistent elevation of these factors in HGSOC compared with normal ovary and fallopian tube (FT) tissues (Figure 1A), with protein‑level overexpression confirmed by CPTAC proteomic data—most notably for SNRPF and SNRPG (data for SNRPB were unavailable) (Figure 1B). Among these, SNRPF was of particular interest due to emerging evidence of tumorigenic potential, yet its role in OC remained undefined.

Integrative analysis of TCGA‑GTEx transcriptomics (Figure 1C), CPTAC proteomics (Figure 1D), and the CSIOVDB database (Figure 1E) consistently demonstrated marked SNRPF overexpression in OC. Increased SNRPF expression was observed across major histopathological subtypes—including serous, endometrioid, and clear cell carcinomas (Figure S1A)—and was more pronounced in FIGO stage III compared with stage I tumors (Figure 1F). Subtype‐specific analysis revealed elevated SNRPF levels across proliferative, mesenchymal, immunoreactive, and differentiated molecular subtypes, with the proliferative subtype exhibiting the highest expression (Figure 1G). Pan‑cancer analysis further indicated that SNRPF is upregulated in the majority of tumor types compared with matched normal tissues (Figure 1H).

To experimentally validate these bioinformatic results, we assessed SNRPF mRNA levels in fresh‑frozen HGSOC and normal ovary/FT tissues using qPCR (Figure 1I) and Western blotting (Figure 1J,K), confirming significant upregulation in tumor samples. Immunohistochemistry (IHC) staining further revealed stronger SNRPF staining in OC tissues compared with normal FT (Figure 1L). Kaplan‐Meier overall survival (OS) (Figure 1M) and progression‐free survival (PFS) (Figure 1N) analyses demonstrated that high SNRPF expression correlated significantly with reduced patient survival. To systematically evaluate the clinical relevance of SNRPF, we further analyzed the comprehensive clinicopathological characteristics of our tissue microarray (TMA) cohort (n = 105). Correlation analysis revealed that high SNRPF protein expression was significantly associated with advanced FIGO stage (*p* = 0.023) and elevated CA‐125 levels (*p* = 0.025) (Table S1). Subsequent univariable Cox regression analyses identified high SNRPF expression as a significant risk predictor for both poor PFS (HR = 1.68, 95% CI: 1.09–2.59, *p* = 0.019) and OS (HR = 1.77, 95% CI: 1.14–2.75, *p* = 0.011) (Tables S2 and S3), alongside other adverse parameters such as advanced FIGO stage and omental involvement. Although multivariable analysis identified FIGO stage and omental involvement as primary independent prognostic factors, the loss of SNRPF independent significance is likely due to its strong collinearity with these advanced clinicopathological features. Nonetheless, the univariable analysis and clinical correlations underscore the robust association of SNRPF overexpression with an aggressive OC phenotype.

Collectively, these multi‑platform transcriptomic, proteomic, and clinical cohort findings establish SNRPF as consistently overexpressed in OC, serving as a reliable indicator of advanced malignant features and adverse patient outcomes.

### 2.2 SNRPF Silencing Inhibits the Proliferative and Metastatic Properties of OC Cells

**FIGURE 2:**
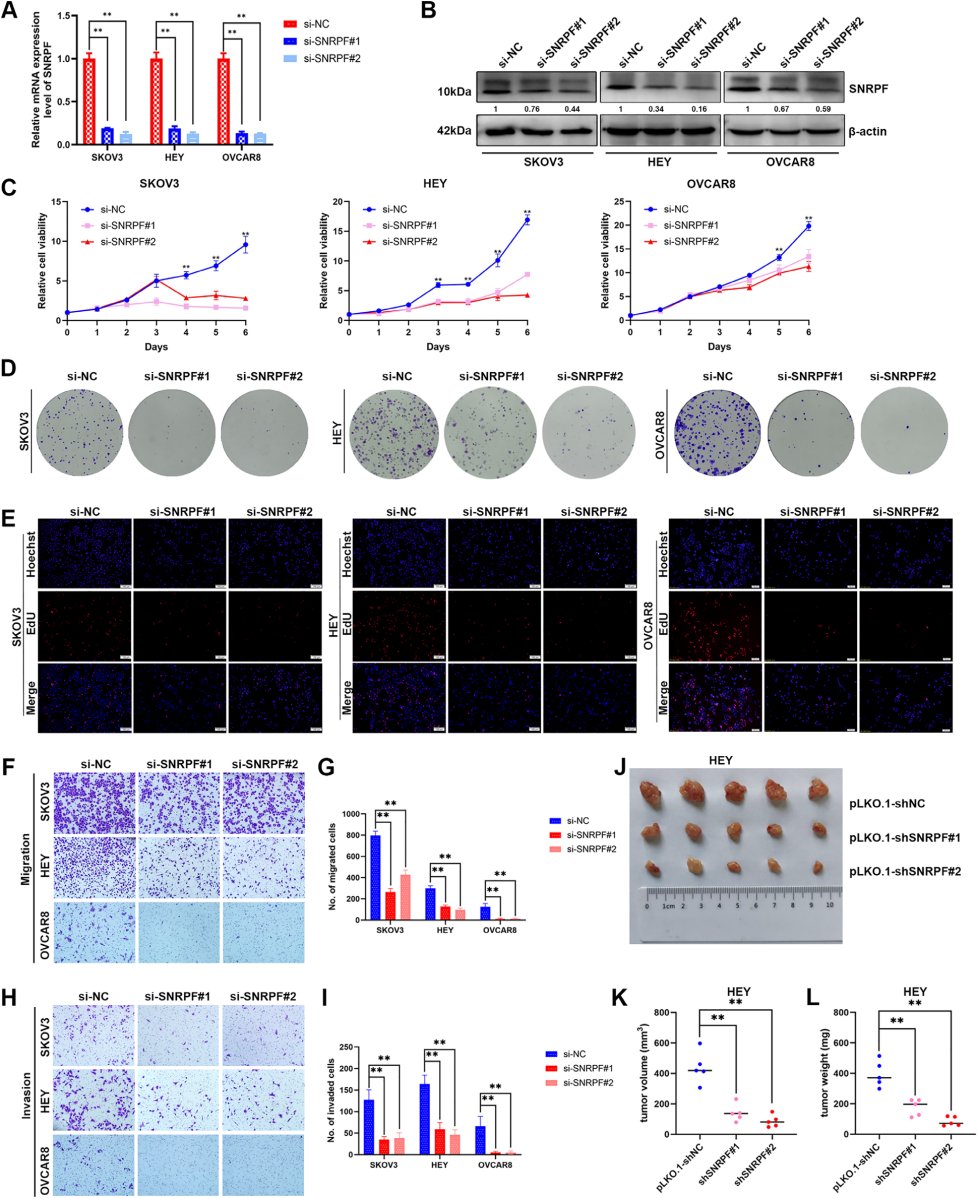
SNRPF Silencing Inhibits the Proliferative and Metastatic Properties of OC Cells. (A) qPCR detection of SNRPF mRNA levels in SKOV3, HEY, and OVCAR8 cells after transient transfection with two independent siRNAs. (B) Western blotting analysis of SNRPF protein levels following siRNA‐mediated knockdown in SKOV3, HEY, and OVCAR8 cells. The numbers below the bands represent relative protein expression levels quantified by densitometry and normalized to β‐actin. (C) MTT assay evaluating the effect of SNRPF depletion on cell proliferation. (D) Representative images of colony formation assays in SNRPF‐silenced cells compared to controls. Corresponding statistical quantification is provided in Figure S1B. (E) Representative images of EdU incorporation assays showing DNA synthesis in SNRPF‐silenced OC cells. Corresponding statistical quantification is provided in Figure S1C. (F) Representative images of Transwell migration assays in SKOV3, HEY, and OVCAR8 cells after SNRPF knockdown. (G) Statistical quantification of migrated cells derived from (F). (H) Representative images of Transwell invasion assays in SKOV3, HEY, and OVCAR8 cells after SNRPF knockdown. (I) Statistical quantification of invaded cells derived from (H). (J) Representative images of subcutaneous tumors obtained from a nude mouse xenograft model using HEY cells with stable shRNA‐mediated SNRPF knockdown (pLKO.1‐shSNRPF#1, pLKO.1‐shSNRPF#2) or corresponding control (pLKO.1‐shNC). (K) Tumor volume comparison among HEY cell‐derived xenografts from pLKO.1‐shSNRPF#1 (*n* = 5), pLKO.1‐shSNRPF#2 (*n* = 5), and pLKO.1‐shNC (*n* = 5) groups, calculated from length and width measurements. (L) Tumor weight comparison among HEY cell‐derived xenografts from pLKO.1‐shSNRPF#1 (*n* = 5), pLKO.1‐shSNRPF#2 (*n* = 5), and pLKO.1‐shNC (*n* = 5) groups after harvest. Statistical significance was determined using two‐way ANOVA followed by Tukey's multiple comparisons test for (C), and one‐way ANOVA followed by Dunnett's multiple comparisons test for quantitative data in (A, G, I, K, and L). ^**^ *p* < 0.01.

To investigate the biological significance of SNRPF in OC, we assessed cellular phenotypic alterations following its knockdown. Transient silencing of SNRPF was achieved using two independent small interfering RNAs (siRNAs) in the SKOV3, HEY, and OVCAR8 cell lines. Successful knockdown of SNRPF expression at both the mRNA and protein levels was confirmed by qPCR (Figure 2A) and Western blotting (Figure 2B) analyses.

Functional evaluation through MTT assays demonstrated that SNRPF depletion significantly inhibited OC cell proliferation (Figure 2C). Consistent with these results, the colony formation capacity markedly decreased upon SNRPF knockdown (Figure 2D and Figure S1B). Moreover, EdU incorporation assays revealed a decrease in the proportion of DNA‐synthesizing cells following SNRPF silencing in OC cells (Figure 2E and Figure S1C). The migration (Figure 2F,G) and invasion (Figure 2H,I) of SKOV3, HEY, and OVCAR8 cells were also suppressed after SNRPF knockdown, as shown by Transwell migration and invasion assays.

To extend these findings in vivo, we employed a nude mouse xenograft model by subcutaneously injecting HEY cells expressing either stable shRNA‐mediated SNRPF knockdown or a corresponding control into the axilla. After 15 days under SPF conditions, tumor specimens were harvested following euthanasia (Figure 2J). Compared with control tumors, tumors derived from SNRPF‐silenced HEY cells exhibited significantly reduced volume (Figure 2K) and weight (Figure 2L).

Collectively, these results substantiate the pivotal role of SNRPF in promoting the malignant behavior of OC cells. Given the established link between spliceosome dysregulation and oncogenic splicing events, we next sought to determine the molecular pathways and specific AS mechanisms—particularly IR—regulated by SNRPF in OC.

### 2.3 Identification of SNRPF‐Regulated Downstream Genes and Splicing Events in OC Cells

**FIGURE 3:**
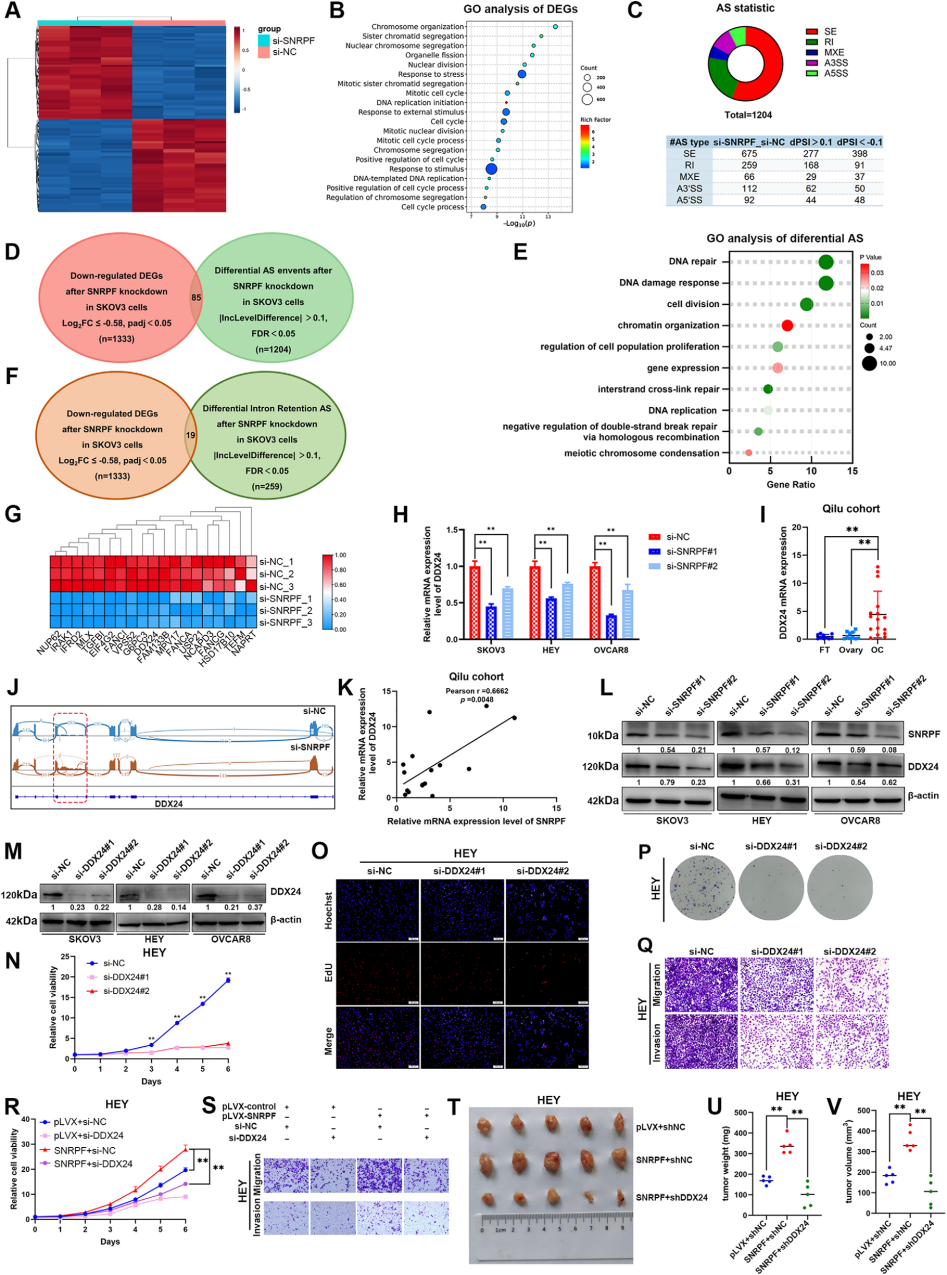
Identification of SNRPF‐Associated Transcriptional and Splicing Alterations, and Functional Characterization of DDX24 in OC. (A) Heatmap visualization of differentially expressed genes identified by RNA‐seq following transient silencing of SNRPF in SKOV3 cells, using the thresholds |log_2_FC| ≥ 1 and padj < 0.05. (B) GO enrichment analysis of the DEGs after SNRPF silencing in SKOV3 cells. (C) Summary of AS events detected using rMATS software, applying |IncLevelDifference| > 0.1 and FDR < 0.05 as cutoffs. (D) Venn diagram illustrating overlap between downregulated DEGs (log_2_FC ≤ ‐0.58, padj < 0.05) and genes showing significant AS changes (|IncLevelDifference| > 0.1, FDR < 0.05) after SNRPF knockdown. (E) GO enrichment analysis of the overlapping gene set identified in panel (D). (F) Comparative analysis of genes with both downregulated expression and significant IR events following SNRPF silencing, identifying 19 candidate genes. (G) Heatmap representation of the 19 candidate genes identified in panel (F). (H) qPCR detection of DDX24 mRNA levels in SKOV3, HEY, and OVCAR8 cells after SNRPF knockdown. (I) qPCR detection of DDX24 mRNA levels in fresh‐frozen OC (*n* = 16), normal ovary (*n* = 10), and normal FT (*n* = 10) tissues. (J) IGV Sashimi plot showing IR in the DDX24 transcript in SKOV3 cells transfected with SNRPF siRNA. (K) Correlation analysis between SNRPF and DDX24 expression in fresh‐frozen OC (*n* = 16), as determined by qPCR. (L) Western blotting analysis of DDX24 protein levels in SKOV3, HEY, and OVCAR8 cells after SNRPF knockdown. The numbers below the bands represent relative protein expression levels quantified by densitometry and normalized to β‐actin. (M) Western blotting analysis of DDX24 expression in SKOV3, HEY, and OVCAR8 cells after transient transfection with specific siRNAs targeting DDX24. The numbers below the bands represent relative protein expression levels quantified by densitometry and normalized to β‐actin. (N) MTT assay to assess cell proliferation following DDX24 knockdown in HEY cells. (O) Representative images of EdU incorporation assays showing DNA synthesis activity in HEY cells after DDX24 silencing. Corresponding statistical quantification of EdU‐positive cells is provided in Figure S2D. (P) Representative images of colony formation assays in HEY cells following DDX24 knockdown. Corresponding statistical quantification is provided in Figure S2F. (Q) Representative images of Transwell migration (top) and invasion (bottom) assays in HEY cells after DDX24 depletion. Corresponding statistical quantification is provided in Figure S2H,J. (R) MTT assay assessing proliferation in HEY cells overexpressing SNRPF with or without transfection of DDX24 siRNAs. (S) Representative images of Transwell assays evaluating the rescue effect of DDX24 knockdown on SNRPF‐overexpressing HEY cells. Corresponding statistical quantification is provided in Figure S2M,N. (T) Representative images of subcutaneous xenograft tumors from nude mice implanted with HEY cells (groups: pLVX+shNC, SNRPF+shNC, SNRPF+shDDX24; *n* = 5 per group). (U) Tumor weight measurements from the HEY xenograft models are shown in panel (T). (V) Tumor volume measurements from the HEY xenograft models are shown in panel (T). Statistical significance was determined using Pearson correlation analysis for (K). For multi‐group comparisons, one‐way ANOVA followed by Dunnett's test was used for (H), while one‐way ANOVA followed by Tukey's test was used for (I, U, and V). Two‐way ANOVA followed by Tukey's test was applied for (N and R). ^**^ *p* < 0.01.

To elucidate the molecular mechanisms underlying the oncogenic role of SNRPF in OC, we conducted RNA Sequencing (RNA‐seq) on SKOV3 cells subjected to SNRPF knockdown via siRNA and compared them to control siRNA‐transfected cells. Differential expression analysis identified 1215 DEGs, including 772 upregulated and 443 downregulated genes upon SNRPF silencing (|log_2_FC| ≥ 1, q < 0.05). Heatmaps illustrating global expression patterns are shown in Figure 3A. Gene Ontology (GO) enrichment analysis revealed strong associations with biological processes such as chromosome organization, sister chromatid segregation, nuclear chromosome segregation, mitotic cell cycle progression, and initiation of DNA replication (Figure 3B), suggesting that SNRPF may promote tumor progression by regulating mitotic and DNA replication pathways.

We next evaluated the impact of SNRPF silencing on the AS landscape using rMATS with stringent criteria (|IncLevelDifference| > 0.1, FDR < 0.05). This analysis identified 1204 significant differential AS events, with exon skipping (ES) being the predominant type (56.06%), followed by IR (21.51%), mutually exclusive exons (MXE; 5.48%), and other subtypes (16.95%) (Figure 3C).

To identify key oncogenic effectors positively regulated by SNRPF, we sought to identify downstream targets subject to dual regulation at both transcriptional and post‐transcriptional levels. To achieve a robust intersection, we compared downregulated genes with those exhibiting significant AS alterations. Notably, applying a conventional threshold of log_2_FC ≤ ‐1 yielded a limited overlap of only 30 genes, which precluded comprehensive pathway analysis. Consequently, we adopted a more inclusive threshold of log_2_FC ≤ −0.58 (corresponding to a 1.5 fold decrease). This adjusted strategy allowed us to capture a broader spectrum of biologically relevant targets (*n* = 85) without compromising statistical validity (Figure 3D). GO enrichment analysis of these 85 candidates revealed a significant involvement in processes critical for genomic stability and cell cycle progression, including DNA repair, DNA damage response, chromatin organization, DNA replication, and meiotic chromosome condensation (Figure 3E).

Given that aberrant IR is known to contribute to tumorigenesis by disrupting tumor suppressor function, activating oncogenes, and altering cell proliferation [18, 19], we refined our analysis to focus specifically on IR‐associated genes. Intersecting downregulated DEGs with genes exhibiting significant IR changes upon SNRPF knockdown identified 19 core candidates (Figure 3F). The expression profiles of these 19 genes following SNRPF silencing are shown in Figure 3G.

To prioritize targets for further functional characterization, we examined IGV‐generated Sashimi plots to validate the IR events of the 19 initial candidate genes. This analysis confirmed clear IR alterations in nine genes: DDX24, NUP62, MPV17, FAM133B, TGFBI, EIF4G2, IFRD2, FANCI, and TEFM. To identify the primary downstream effector, we performed a comparative experimental screening of these nine candidates. We systematically evaluated their mRNA expression via qPCR following SNRPF knockdown with two independent siRNAs in SKOV3, HEY, and OVCAR8 cell lines (Figure S1D–F. Strikingly, DDX24 was the only candidate that exhibited consistent and significant downregulation across all tested cell lines and siRNA treatments (Figure 3H). Clinical validation using qPCR on patient samples revealed significantly elevated DDX24 mRNA levels in OC tissues compared to normal ovary and FT controls (Figure 3I). Consequently, DDX24 was identified as the primary downstream target of SNRPF for subsequent investigation.

Specifically, IGV visualization highlighted increased retention of intron 6 within the DDX24 transcript following SNRPF depletion (Figure 3J). Furthermore, Pearson correlation analysis demonstrated a robust positive association between SNRPF and DDX24 expression in these clinical specimens (r  =  0.6662) (Figure 3K). Finally, Western blotting analysis confirmed that SNRPF depletion markedly reduced DDX24 protein abundance in SKOV3, HEY, and OVCAR8 cells (Figure 3L).

Collectively, these data establish DDX24 as a critical downstream effector whose expression is regulated by SNRPF via IR‐mediated splicing regulation in OC.

### 2.4 DDX24 Promotes Malignant Phenotypes and Mediates SNRPF‐Driven Oncogenesis in OC Cells

To investigate the functional role of DDX24 in OC progression, we performed a series of loss‐of‐function assays. Specific siRNAs were employed to silence DDX24 expression in SKOV3, HEY, and OVCAR8 cells (Figure 3M and Figure S2A). Cell proliferation assays demonstrated that DDX24 knockdown significantly inhibited the growth of these OC cells (Figure 3N and Figure S2B). This was further supported by EdU incorporation assays, which showed a marked decrease in DNA synthesis following DDX24 depletion (Figure 3O and Figure S2C,D). Consistently, colony formation assays revealed that DDX24 silencing compromised the long‐term proliferative capacity of all three cell lines (Figure 3P and Figure S2E,F). Additionally, Transwell migration and invasion assays demonstrated that DDX24 knockdown significantly impaired the metastatic potential of OC cells (Figure 3Q and Figure S2G–J).

To determine whether the SNRPF‐mediated oncogenic effects are dependent on DDX24, we performed abrogation experiments by silencing DDX24 in SNRPF‐overexpressing cells. Consistent with our hypothesis, DDX24 silencing significantly attenuated the increases in cell proliferation (Figure 3R), clonogenicity (Figure S2K,L), and metastatic potential (Figure 3S and Figure S2M,N) induced by ectopic SNRPF expression. To validate this SNRPF/DDX24 axis in vivo, we utilized a subcutaneous xenograft model. The results showed that DDX24 suppression effectively reversed the accelerated tumor growth driven by SNRPF overexpression, as evidenced by representative xenograft images (Figure 3T) and quantitative measurements of tumor volume and weight (Figure 3U,V).

To further substantiate this functional dependency, we conducted reciprocal rescue experiments by ectopically expressing DDX24 in SNRPF‐depleted cells. Notably, the impairments in cell proliferation (Figure S2O), clonogenicity (Figure S2P,Q), and metastatic potential (Figure S2R,T) resulting from SNRPF knockdown were significantly restored by DDX24 restoration. Collectively, these bidirectional functional assays—encompassing both gain‐ and loss‐of‐function models—identify DDX24 as a critical downstream mediator of SNRPF‐driven progression in OC.

### 2.5 SNRPF Modulates Intron 6 Retention in DDX24 Transcripts in OC Cells

**FIGURE 4:**
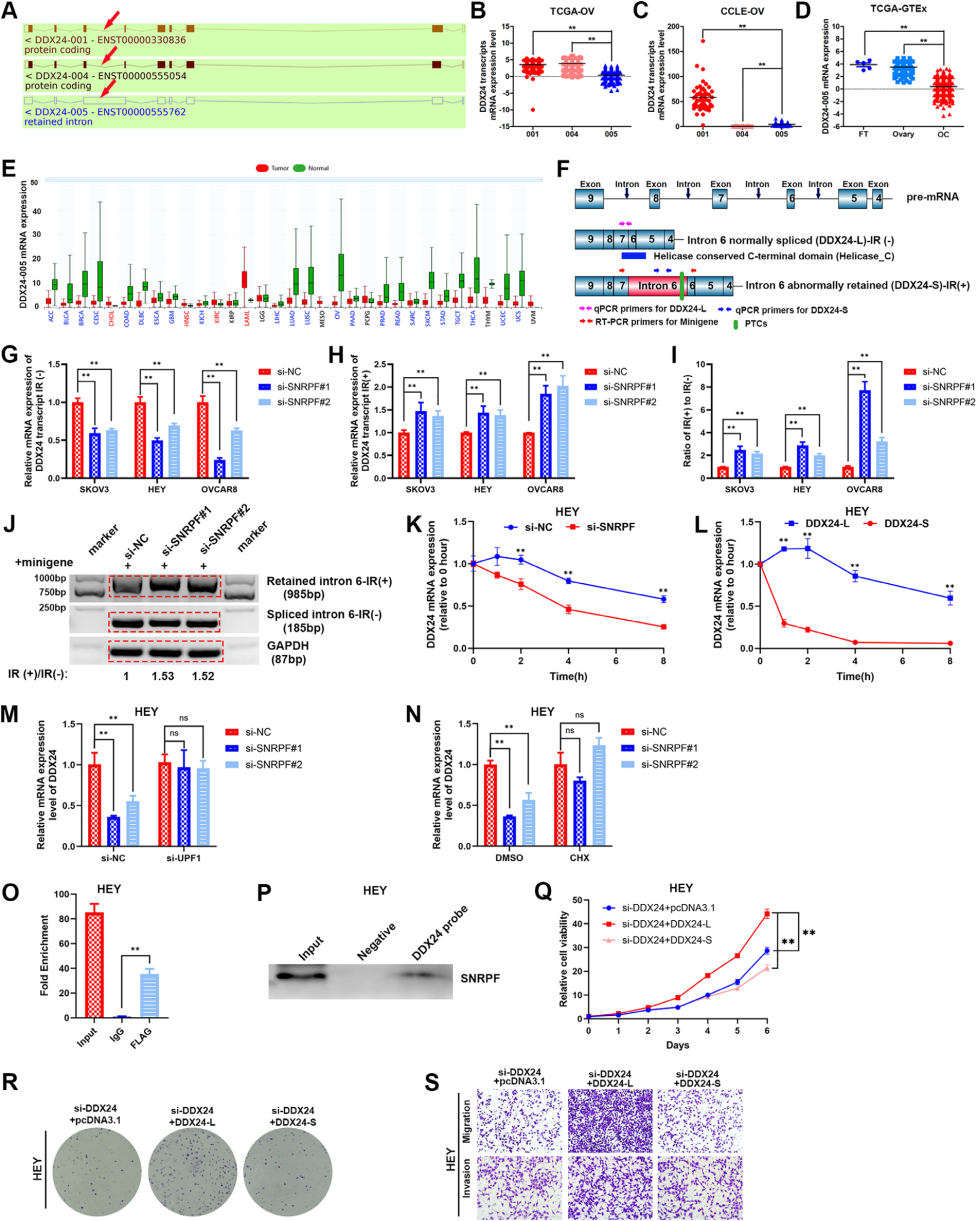
SNRPF Modulates Intron 6 Retention in DDX24 Transcripts in OC Cells. (A) Overview of annotated DDX24 transcript variants obtained from the Ensembl database. (B) Transcript abundance analysis of DDX24‐001, DDX24‐004, and DDX24‐005 using RNA‐seq datasets from the TCGA‐OV cohort (*n* = 426). (C) Isoform‐specific expression levels of DDX24 transcripts in OC cell lines (*n* = 47) from the CCLE database. (D) Comparative transcript analysis of DDX24‐005 across OC (*n* = 426, TCGA), normal ovary (*n* = 88, GTEx), and FT (*n* = 5, GTEx) tissues. (E) Pan‐cancer analysis of DDX24‐005 transcript levels using TCGA datasets, comparing tumor and matched normal tissues. (F) Schematic representation of qPCR primer locations designed to distinguish among DDX24 transcript isoforms. (G‐H) qPCR detection of normally spliced (DDX24‐L, IR‐) and intron 6‐retained (DDX24‐S, IR+) transcript variants in OC cells after SNRPF silencing. (I) Alteration of the DDX24‑S/DDX24‑L isoform ratio in OC cells upon SNRPF knockdown. (J) RT‐PCR analysis of DDX24 transcript variants using a minigene construct containing partial exon 5, exon 6, truncated intron 6 (including ∼500 bp flanking the splice sites), and exon 7, cotransfected with SNRPF‐targeting siRNA in HEY cells. Densitometric quantification of the DDX24‐S to DDX24‐L ratio was performed using ImageJ. (K) Analysis of DDX24 transcript stability following actinomycin D treatment in SNRPF‐knockdown HEY cells. (L) Comparative stability assessment of intron‐retained (DDX24‐S) and normally spliced (DDX24‐L) transcripts after actinomycin D exposure in HEY cells. (M) qPCR detection of DDX24 mRNA levels in SNRPF‐depleted HEY cells after UPF1 silencing. (N) qPCR detection of DDX24 mRNA levels in SNRPF‐depleted HEY cells following CHX treatment. (O) RIP analysis of DDX24 mRNA using anti‐Flag antibody in HEY cells overexpressing Flag‐tagged SNRPF. (P) RNA pull‐down assays using biotin‐labeled probes targeting intron 6 of DDX24 in HEY cells, followed by Western blotting to detect SNRPF. Biotinylated antisense transcripts were used as a negative control. (Q) MTT assay assessing cell proliferation in cells transfected with pcDNA3.1 vector, pcDNA3.1‐DDX24‐L, or pcDNA3.1‐DDX24‐S after endogenous DDX24 depletion. (R) Representative images of colony formation assays evaluating clonogenic capacity in cells treated as described in panel (Q). Corresponding statistical quantification is provided in Figure S3B. (S) Representative images of Transwell migration and invasion assays in cells treated as described in panel (Q). Corresponding statistical quantification is provided in Figure S3C,D. Statistical significance was determined using Student's *t*‐test (O); one‐way ANOVA followed by Dunnett's test (G, H, and I); one‐way ANOVA followed by Tukey's test (B, C, D); and two‐way ANOVA followed by Tukey's test (K, L, M, N, and Q). ^**^ *p* < 0.01; ns, not significant.

To investigate the structural variations of DDX24, we analyzed annotated isoforms from the Ensembl database. The protein‐coding isoforms DDX24‐001 (ENST00000330836, 859 aa) and DDX24‐004 (ENST00000555054, 816 aa) lack intron 6, whereas DDX24‐005 (ENST00000555762) retains intron 6, introduces PTCs, and disrupts protein synthesis (Figure 4A). TCGA‐OV transcriptomic data showed significantly higher expression of the protein‐coding DDX24‐001 and DDX24‐004 isoforms compared to the intron‐retained DDX24‐005 transcript in OC tissues (Figure 4B). CCLE database analysis confirmed DDX24‐001 as the most abundantly expressed isoform across OC cell lines (Figure 4C). Furthermore, DDX24‐005 was significantly downregulated in OC relative to normal ovary and FT samples based on TCGA‐GTEx data (Figure 4D), a pattern consistent across multiple tumor types in pan‐cancer analysis (Figure 4E).

To experimentally assess isoform‐specific changes, we designed qPCR primers spanning the exon 6–exon 7 junction/exon 7 (detecting the normally spliced long isoform, DDX24‐L) and primers targeting the retained intron 6 (detecting the short intron‐retained isoform, DDX24‐S) (Figure 4F). Functionally, DDX24 contains a conserved C‐terminal helicase domain (Helicase_C) encoded by portions of exons 5, 6, and 7, which is essential for its ATP‐dependent RNA binding and unwinding activity [20, 21]. Notably, the retention of intron 6 introduces PTCs, which are predicted to disrupt the integrity of this critical Helicase_C domain.

As hypothesized, SNRPF knockdown significantly reduced DDX24‐L (IR‐) levels (Figure 4G) while increasing DDX24‐S (IR+) abundance (Figure 4H), resulting in an elevated DDX24‐S/DDX24‐L ratio (Figure 4I). RT‐PCR using minigene constructs containing exon 5, exon 6, truncated intron 6, and exon 7 confirmed this splicing shift upon SNRPF depletion in HEY cells (Figure 4J).

Transcript stability assays using Actinomycin D revealed that SNRPF knockdown reduced the half‐life of DDX24 transcripts (Figure 4K), with DDX24‐S displaying notably lower stability than DDX24‐L in HEY cells (Figure 4L). Because intron 6 retention introduces PTCs, these aberrant transcripts are predicted to be degraded via NMD. Indeed, UPF1 silencing restored DDX24 levels in SNRPF‐depleted HEY cells (Figure 4M), and treatment with the NMD inhibitor cycloheximide (CHX) yielded similar recovery in HEY cells (Figure 4N). This rapid NMD‐mediated clearance accords with our earlier observations (Figure 3H,L) that SNRPF depletion substantially limits total DDX24 mRNA and functional protein levels without generating detectable truncated DDX24‐S protein.

To examine the physical interaction between SNRPF and DDX24 transcripts, RNA immunoprecipitation (RIP) assays using an anti‐Flag antibody were performed in SNRPF‐overexpressing HEY cells, which showed significant enrichment of DDX24 mRNA in SNRPF‐associated complexes (Figure 4O). Furthermore, RNA pull‐down assays using an in vitro transcribed, biotinylated probe spanning the exon 6–intron 6–exon 7 junction demonstrated that the biotinylated DDX24 intron 6 sequence successfully precipitated endogenous SNRPF protein, whereas the antisense control did not (Figure 4P). These results provide direct molecular evidence that SNRPF physically recognizes and binds to the specific IR site of DDX24, confirming a direct interaction at the site of IR.

Because the endogenous DDX24‐S transcript is rapidly degraded by NMD, we engineered an artificial expression system to evaluate its intrinsic protein stability. Specifically, we constructed 3×Flag‐tagged expression vectors where, for DDX24‐S, the native sequence downstream of the PTC was excluded and the truncated coding sequence was directly fused to the 3×Flag tag. This design effectively bypassed endogenous NMD surveillance. Notably, even under these forced overexpression conditions, the translated DDX24‐S truncated protein proved highly unstable, presenting as a substantially weaker band compared to DDX24‐L (Figure S3A). Following endogenous DDX24 silencing, overexpression of DDX24‐L in HEY cells significantly promoted cell proliferation (Figure 4Q), clonogenicity (Figure 4R and Figure S3B), and metastatic potential (Figure 4S and Figure S3C,D), whereas DDX24‐S showed minimal oncogenic activity. This lack of activity in DDX24‐S likely stems from the disruption of the Helicase_C domain identified earlier.

In summary, SNRPF ensures proper intron 6 splicing of DDX24 to maintain the expression of its working isoforms. Loss of SNRPF induces IR, generating PTC‐containing transcripts that are eliminated via NMD, thereby abrogating the oncogenic activity of DDX24 in OC.

### 2.6 Identification and Validation of DDX24 Downstream Effectors in OC Cells

**FIGURE 5:**
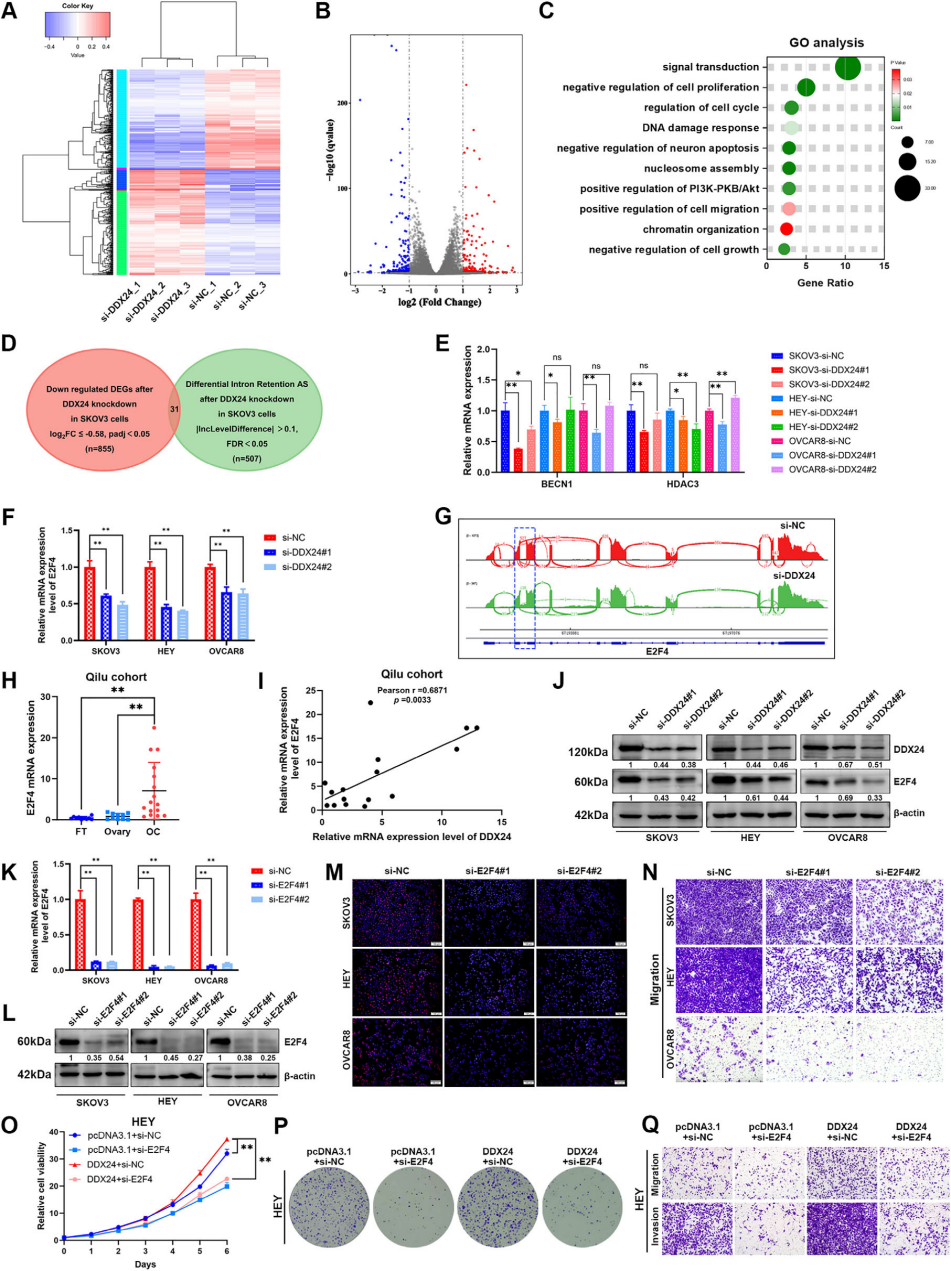
Identification of DDX24‐Regulated Downstream Effectors and Functional Characterization of E2F4 in OC Cells. (A‐C) Heatmap representation (A), volcano plot (B), and GO enrichment analysis (C) of DEGs identified by RNA‐seq in SKOV3 cells transfected with DDX24‐specific siRNAs or control siRNA. (D) Venn diagram showing the overlap between downregulated DEGs (log_2_FC ≤ ‐0.58; consistent with the SNRPF analysis parameters) and genes with significant IR events (|IncLevelDifference| > 0.1, FDR < 0.05) upon DDX24 silencing. (E‐F) qPCR analysis of BECN1, HDAC3 (E), and E2F4 (F) mRNA levels in SKOV3, HEY, and OVCAR8 cells after DDX24 knockdown. (G) IGV Sashimi plot displaying intron 2 retention in the E2F4 transcript after DDX24 silencing in SKOV3 cells. The boxed area highlights the increased reads in the retained intron region. (H) qPCR detection of E2F4 mRNA levels in the Qilu cohort, comprising fresh‐frozen OC (*n* = 16), normal ovary (*n* = 10), and normal FT (*n* = 10) tissues. (I) Pearson correlation analysis between DDX24 and E2F4 mRNA expression in the Qilu cohort (*n* = 16). (J) Western blotting analysis of E2F4 protein levels in OC cells after DDX24 knockdown. The numbers below the bands represent relative protein expression levels quantified by densitometry and normalized to β‐actin. (K‐L) qPCR (K) and Western blotting (L) verification of E2F4 knockdown efficiency in OC cells transfected with specific siRNAs. The numbers below the bands represent relative protein expression levels quantified by densitometry and normalized to β‐actin. (M, N) Representative images of EdU incorporation assays (M) and Transwell migration assays (N) in OC cells following transient E2F4 silencing. Corresponding statistical quantifications are provided in Figure S4D,E. (O) MTT assay assessing cell viability in HEY cells overexpressing DDX24 with or without co‐transfection of E2F4 siRNAs. (P‐Q) Representative images of colony formation (P) and Transwell migration/invasion assays (Q) in HEY cells treated as described in panel (O). Corresponding statistical quantification is provided in Figure S4H–J. Data are presented as the mean ± SD from three independent experiments. Statistical significance was determined using one‐way ANOVA followed by Dunnett's test (E, F, and K), one‐way ANOVA followed by Tukey's test (H), Pearson correlation analysis (I), and two‐way ANOVA Tukey's multiple comparisons test (O). ^*^ *p* < 0.05, ^**^ *p* < 0.01; ns, not significant.

Given the oncogenic role of DDX24 and its regulation by SNRPF‐mediated splicing, we next sought to identify downstream effectors through which DDX24 promotes OC malignancy. RNA sequencing was performed on SKOV3 cells transfected with either DDX24‐targeting siRNAs or control siRNA. Differential expression analysis identified 440 DEGs (225 upregulated and 215 downregulated genes; |log_2_FC| ≥ 1, padj < 0.05), as visualized in the heatmap (Figure 5A) and volcano plot (Figure 5B). GO enrichment analysis indicated that these DEGs were predominantly involved in processes such as signal transduction, regulation of cell proliferation and cycle progression, DNA damage response, cell migration and growth, and the PI3K‐PKB/Akt signaling pathway (Figure 5C).

Given the critical importance of IR in dictating transcript stability during cancer progression, we performed an integrated analysis to identify direct splicing targets. To comprehensively capture potential candidates, we mapped downregulated DEGs utilizing a slightly relaxed transcriptomic threshold (log_2_FC ≤ −0.58; consistent with the previous SNRPF analysis parameters) against genes exhibiting significant IR alterations (|IncLevelDifference| > 0.1, FDR < 0.05) in DDX24‐depleted cells. This intersection identified 31 potential candidates regulated via aberrant IR (Figure 5D). To prioritize the most robust candidates for functional validation, we re‐applied the stringent transcriptional threshold (log_2_FC ≤ ‐1) to this subset, which narrowed the list to three primary candidates: E2F4, HDAC3, and BECN1.

To elucidate the bona fide downstream effector, we performed comparative validation of these three candidates in multiple OC cell lines (SKOV3, HEY, and OVCAR8) using two independent siRNAs. qPCR analysis revealed that HDAC3 and BECN1 exhibited variable suppression that was highly cell line‐ or siRNA‐dependent (Figure 5E). In contrast, E2F4 mRNA levels were consistently and significantly reduced across all cell lines and siRNA treatments (Figure 5F). Consequently, E2F4 was selected for detailed mechanistic study based on its robust, context‐independent regulation by DDX24 and its established oncogenic role.

Visualization of RNA‐seq data via IGV Sashimi plots confirmed pronounced retention of intron 2 in E2F4 transcripts upon DDX24 silencing (Figure 5G), suggesting that DDX24 modulates E2F4 expression through splicing regulation. Expression analysis in our fresh‐frozen clinical cohort demonstrated significantly higher E2F4 mRNA levels in OC tissues compared to normal ovary and FT specimens (Figure 5H). Pearson correlation analysis further revealed a strong positive association between DDX24 and E2F4 expression in OC samples (r  =  0.6871) (Figure 5I).

We further verified this regulatory axis at the protein level. Western blotting results consistently demonstrated a significant decrease in E2F4 protein abundance (Figure 5J) following DDX24 depletion, supporting the notion that DDX24 sustains E2F4 expression. To further confirm the dependency of this regulation within the SNRPF‐driven axis, we overexpressed SNRPF in cells with or without DDX24 knockdown. In control cells (HEY and SKOV3), ectopic SNRPF expression upregulated E2F4 protein levels (Figure S3E,F). However, this stimulatory effect was effectively abrogated upon DDX24 silencing (Figure S3G,H), indicating that DDX24 is an essential mediator for SNRPF‐facilitated E2F4 expression.

Collectively, these results identify E2F4 as a critical downstream effector through which DDX24—under the control of SNRPF‐mediated splicing—facilitates OC progression. This finding delineates a hierarchical cascade: SNRPF ensures proper DDX24 splicing, which in turn maintains E2F4 expression by repressing its aberrant IR, thereby driving malignant behaviors.

### 2.7 Oncogenic Activity of E2F4 in OC Progression

To investigate the role of E2F4 in OC cell proliferation and metastasis, we performed a series of in vitro experiments. Using specific siRNAs, we transiently knocked down E2F4 expression levels in multiple OC cell lines (Figure 5K,L). MTT assays revealed that E2F4 depletion significantly inhibited OC cell growth (Figure S4A). Consistent with these findings, colony formation assays exhibited a notable decrease in the colony‐forming ability of cells after E2F4 silencing (Figure S4B,C). EdU incorporation assays further confirmed a reduced percentage of SKOV3, HEY, and OVCAR8 cells undergoing DNA synthesis following E2F4 knockdown (Figure 5M and Figure S4D). Additionally, Transwell assays indicated that the reduction in E2F4 expression impaired both the migration (Figure 5N and Figure S4E) and invasion (Figure S4F,G) of these OC cells.

Next, to assess whether E2F4 mediates the oncogenic effects driven by DDX24, we performed bidirectional rescue experiments. First, we conducted abrogation assays in HEY cells where DDX24 was overexpressed. We found that the subsequent silencing of E2F4 by siRNAs markedly attenuated the DDX24‐induced increases in cell growth rate (Figure 5O), colony formation (Figure 5P and Figure S4H), and metastatic potential (Figure 5Q and Figure S4I,J). In a complementary approach, we conducted functional rescue experiments; specifically, the ectopic expression of E2F4 in DDX24‐knockdown cells significantly restored the impaired proliferation (Figure S5A), colony formation (Figure S5B,C), and metastatic capabilities (Figure S5D–F) induced by DDX24 depletion.

Taken together, these bidirectional functional assays firmly demonstrate that E2F4 is a critical downstream mediator executing the DDX24‐driven oncogenic phenotype, promoting OC progression by accelerating cell cycle/proliferation and enhancing metastasis.

### 2.8 DDX24 Facilitates Efficient Splicing of E2F4 to Sustain Its Elevated Expression in OC Cells

**FIGURE 6:**
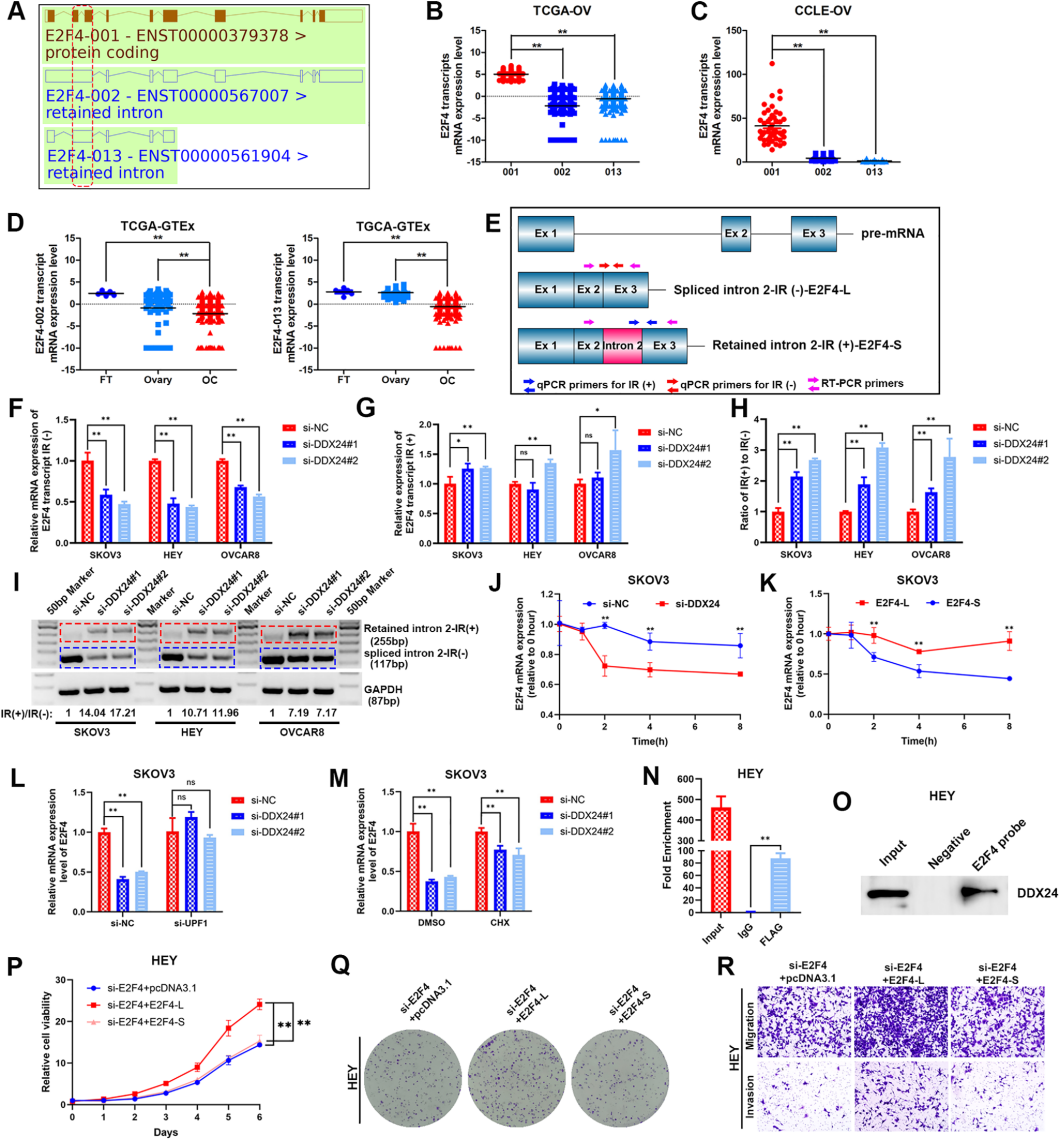
DDX24 Facilitates Efficient Splicing of E2F4 to Sustain Its Elevated Expression in OC Cells. (A) Schematic representation of E2F4 transcript variants (E2F4‐001, E2F4‐002, and E2F4‐013) annotated in the Ensembl database. (B) Transcript abundance of E2F4 isoforms in the TCGA‐OV cohort (*n* = 426). (C) Expression levels of E2F4 isoforms in OC cell lines (*n* = 47) derived from the CCLE database. (D) Comparative expression analysis of intron‐retaining E2F4 isoforms (E2F4‐002 and E2F4‐013) in normal FT (*n* = 5, GTEx), normal ovary (*n* = 88, GTEx), and OC tissues (*n* = 426, TCGA). (E) Schematic illustration of primer locations designed to specifically amplify spliced (IR–) and intron‐retained (IR+) E2F4 transcripts. (F‐G) qPCR quantification of (F) functionally spliced E2F4 transcripts (IR–, corresponding to E2F4‐L) and (G) intron 2‐retained transcripts (IR+, corresponding to E2F4‐S) in OC cells following DDX24 knockdown. (H) Ratio of E2F4‐S to E2F4‐L transcript abundance in OC cells following DDX24 knockdown. (I) Representative RT‐PCR gel images distinguishing E2F4‐L (spliced) and E2F4‐S (retained) transcripts in OC cells transfected with DDX24 siRNAs. Densitometric quantification of the E2F4‐S to E2F4‐L ratio was performed using ImageJ. (J, K) The mRNA stability analysis of total E2F4 (J) and E2F4‐L vs. E2F4‐S isoforms (K) in SKOV3 cells treated with actinomycin D over the indicated time course. (L) qPCR analysis of E2F4 mRNA levels in DDX24‐depleted SKOV3 cells co‐transfected with UPF1 siRNA to assess NMD activity. (M) qPCR analysis of E2F4 mRNA levels in DDX24‐depleted SKOV3 cells treated with CHX. (N) RIP analysis of E2F4 mRNA using an anti‐Flag antibody in HEY cells overexpressing Flag‐tagged DDX24. (O) RNA pull‐down assays using biotin‐labeled probes targeting intron 2 of E2F4 in HEY cells, followed by Western blot analysis to detect DDX24 enrichment. Biotinylated antisense transcripts were used as a negative control. (P) Cell viability determined by MTT assay in HEY cells following endogenous E2F4 silencing and rescue via overexpressing vector control, E2F4‐L, or E2F4‐S. (Q) Representative images of colony formation assays in HEY cells treated as described in panel (P). Corresponding statistical quantification is provided in Figure S6B. (R) Representative images of Transwell migration and invasion assays in HEY cells treated as described in panel (P). Corresponding statistical quantification is provided in Figure S6C,D. Data are presented as the mean ± SD from three independent experiments. Statistical significance was determined using Student's *t*‐test (N), one‐way ANOVA followed by Dunnett's test (F, G, and H), one‐way ANOVA followed by Tukey's test for multi‐group comparisons (B, C, and D), and two‐way ANOVA followed by Tukey's multiple comparisons test (J, K, L, M, and P). ^*^ *p* < 0.05, ^**^ *p* < 0.01; ns, not significant.

Having identified E2F4 as a critical downstream effector of DDX24 in OC cells, we next explored how DDX24 regulates intron 2 retention in E2F4 transcripts. Ensembl annotation revealed that the E2F4‑001 transcript (ENST00000379378) encodes a functional protein of 413 amino acids, whereas the E2F4‑002 (ENST00000567007) and E2F4‑013 (ENST00000561904) transcripts retain intron 2, introduce PTCs, and lack protein‐coding potential (Figure 6A).

Analysis of transcriptomic data from the TCGA‐OV cohort revealed that the E2F4‐001 transcript was expressed at much higher levels in OC samples compared to the intron‐retaining transcripts E2F4‐002 and E2F4‐013 (Figure 6B). These findings were supported by expression data from OC cell lines in the CCLE database, where E2F4‐001 was the dominant variant (Figure 6C). Moreover, both E2F4‐002 and E2F4‐013 were expressed at significantly lower levels in OC tissue samples than in normal FT and ovary tissue samples (Figure 6D). These findings indicate that the protein‑coding E2F4‑001 transcript predominates in OC.

To evaluate the effect of DDX24 depletion on E2F4 transcript levels, we designed variant‐specific qPCR primers. To specifically detect the correctly spliced E2F4 transcript lacking intron 2 (E2F4‐L), the forward primer spanned the exon 2–exon 3 junction, and the reverse primer was located within exon 3. In contrast, intron 2‐retaining E2F4 transcripts (E2F4‐S) were measured using a forward primer within the retained intron 2 and a reverse primer in exon 3. Additionally, to assess E2F4 intron 2 splicing by RT‐PCR after DDX24 knockdown, forward and reverse primers targeting exon 2 and exon 3, respectively, were used. The schematic locations of all the primer pairs are shown in Figure 6E.

Silencing DDX24 significantly reduced the abundance of E2F4‑L (IR­) (Figure 6F) while slightly increasing E2F4‑S (IR+) (Figure 6G) levels, resulting in a pronounced shift toward E2F4‑S accumulation (Figure 6H). RT‑PCR confirmed this splicing change, showing decreased E2F4‑L and increased E2F4‑S after DDX24 knockdown, with a markedly elevated E2F4‑S/E2F4‑L ratio (Figure 6I). These findings indicate that loss of DDX24 impairs normal E2F4 splicing and promotes intron 2 retention.

Because IR can impair mRNA stability via the NMD pathway, we assessed transcript half‐life after transcriptional blockade with actinomycin D. DDX24 knockdown significantly reduced E2F4 mRNA stability (Figure 6J), and E2F4‐S was markedly less stable than E2F4‐L in SKOV3 cells (Figure 6K). Consistent with NMD targeting, UPF1 silencing (Figure 6L) or cycloheximide treatment (Figure 6M) restored E2F4 transcript levels in DDX24‐depleted SKOV3 cells. This rapid NMD‐mediated clearance provides a mechanistic basis for our earlier observations (Figure 5F,J) that DDX24 depletion markedly reduces total E2F4 mRNA and functional protein levels without yielding any detectable endogenous truncated E2F4‐S protein.

To determine whether DDX24 physically interacts with E2F4 transcripts, we performed RIP assays in DDX24‐overexpressing HEY cells. Using an anti‐Flag antibody, we detected a marked enrichment of E2F4 mRNA within DDX24‐associated complexes (Figure 6N). To further investigate whether DDX24 directly regulates E2F4 splicing by binding to its pre‐mRNA, we performed biotinylated RNA pull‐down assays. A biotin‐labeled RNA probe spanning the exon 2–intron 2–exon 3 junctions of the E2F4 pre‐mRNA was incubated with nuclear lysates from HEY cells. Western blot analysis showed that the E2F4 intron 2 probe, but not the antisense control, successfully precipitated endogenous DDX24 protein (Figure 6O). These data confirm that DDX24 directly recognizes and binds to the sequence of E2F4 intron 2, thereby mediating its splicing and subsequent mRNA stability.

Finally, to evaluate isoform‑specific functions, we generated pcDNA3.1 constructs expressing E2F4‑L or E2F4‑S proteins. Because endogenous E2F4‐S transcripts are efficiently cleared by NMD, we utilized 3×Flag‐tagged vectors lacking the native downstream PTC context to bypass NMD and evaluate their intrinsic protein stability. Strikingly, even under these forced overexpression conditions, the translated E2F4‐S truncated protein was barely detectable compared to the robust expression of E2F4‐L (Figure S6A), corroborating its inherent instability. Following siRNA‐mediated silencing of endogenous E2F4, overexpression of E2F4‑L substantially enhanced proliferation (Figure 6P), colony formation (Figure 6Q and Figure S6B), and metastatic capacity (Figure 6R and Figure S6C,D) in HEY cells, whereas E2F4‑S had minimal impact on these phenotypes. These findings further support that the DDX24 deficiency‐induced splicing switch toward the unstable and non‐functional E2F4‐S isoform is a pivotal step in disrupting the SNRPF/DDX24/E2F4 regulatory axis.

Having established this SNRPF/DDX24/E2F4 signaling axis, we next investigated whether its initiation is uniquely dependent on SNRPF or represents a redundant function of the broader Sm protein complex. To this end, we silenced other highly expressed Sm counterparts—specifically SNRPB, SNRPD1, and SNRPG—across three OC cell lines (Figure S6E–G). Unlike the significant downregulation observed upon SNRPF depletion, silencing these homologous proteins failed to consistently alter the mRNA levels of either DDX24 or its downstream effector E2F4 (Figure S6H–J). These results demonstrate that the regulation of the DDX24/E2F4 oncogenic axis is not a non‐specific byproduct of general spliceosome disruption, but is specifically coordinated by SNRPF.

Taken together, these findings demonstrate that DDX24 maintains high E2F4 expression in OC cells by ensuring efficient splicing of intron 2 to generate the protein‐coding E2F4‐L isoform. Loss of DDX24 shifts splicing toward noncoding, NMD‐targeted transcripts, which fail to produce stable truncated proteins, thereby attenuating E2F4‐mediated oncogenic effects. This defines E2F4 as a key splicing‐dependent regulatory node within the SNRPF–DDX24–E2F4 axis driving OC progression.

### 2.9 E2F4 Directly Activates SNRPF Transcription via Promoter Binding in OC Cells

We previously established that DDX24 sustains high E2F4 expression in OC by promoting the splicing of E2F4 transcripts. Based on these findings, we reasoned that E2F4, which functions as a transcription factor, might directly regulate SNRPF expression, potentially forming a positive feedback loop that drives OC progression.

**FIGURE 7:**
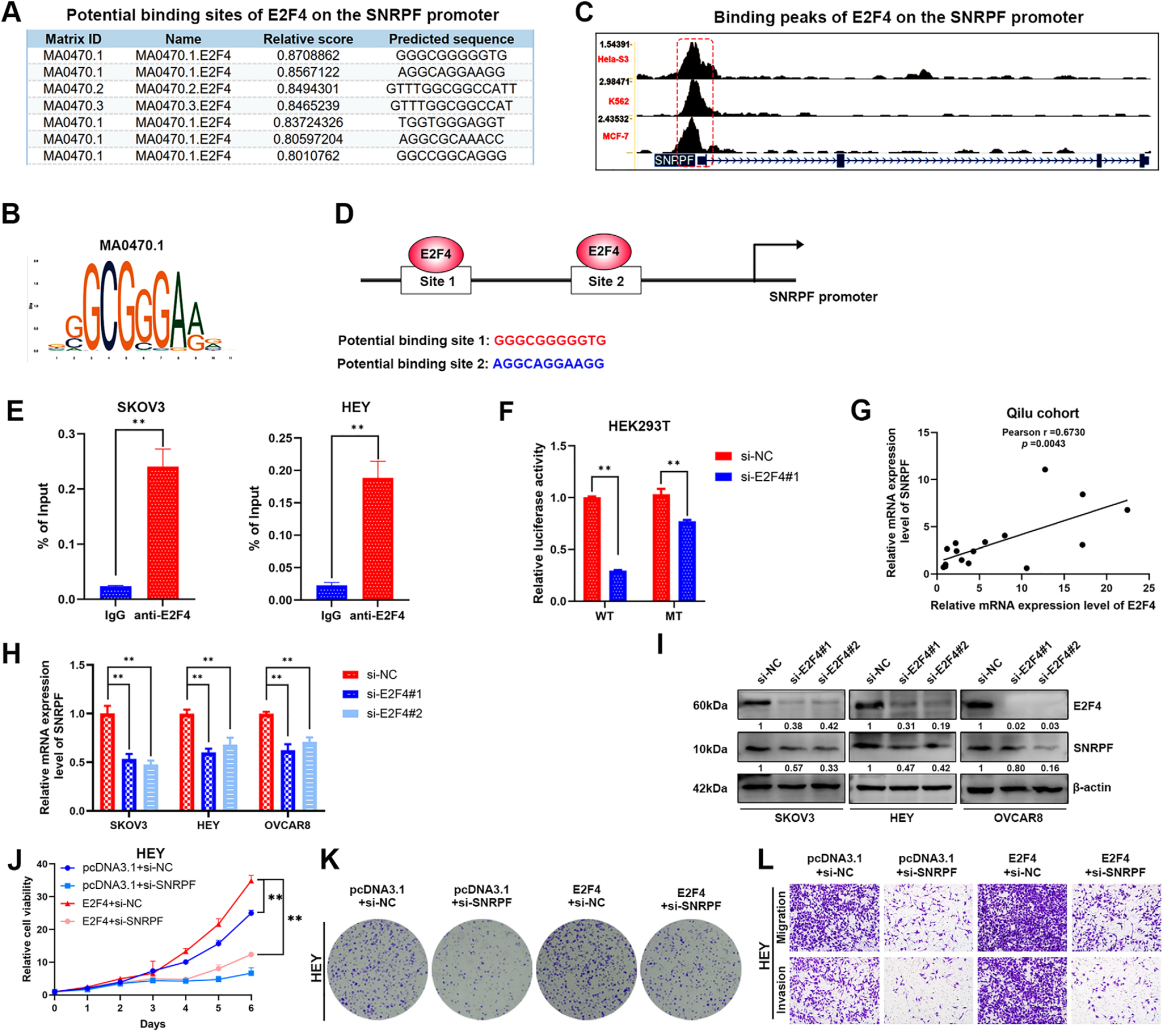
E2F4 Directly Activates SNRPF Transcription via Promoter Binding in OC Cells. (A) Bioinformatic prediction of potential E2F4 binding sites within the SNRPF promoter region predicted by motif scanning analysis. (B) Sequence logo of the consensus E2F4‐binding motif (Matrix ID: MA0470.1) retrieved from the JASPAR database. (C) ChIP‐seq signal tracks displaying E2F4‐binding peaks at the SNRPF promoter across HeLa‐S3, K562, and MCF‐7 cell lines (Data *source*: Cistrome Data Browser). (D) Schematic diagram of the SNRPF promoter region indicating the predicted locations and sequences of potential E2F4‐binding sites 1 and 2. (E) ChIP‐qPCR validation of endogenous E2F4 physical occupancy at the SNRPF promoter in SKOV3 and HEY cells. Data are presented as the percentage of input (% of Input). (F) Dual‐luciferase reporter assays in HEK293T cells. The relative luciferase activity of the wild‐type (WT) or binding‐site‐mutant (MT) SNRPF promoter constructs was assessed following co‐transfection with control (si‐NC) or E2F4‐targeting siRNAs. (G) Pearson correlation analysis of E2F4 and SNRPF mRNA expression levels in the fresh‐frozen OC specimens of the Qilu cohort (*n* = 16). (H) qPCR measurement of SNRPF mRNA levels in SKOV3, HEY, and OVCAR8 cells following E2F4 knockdown. (I) Western blotting analysis of SNRPF protein levels in OC cells following E2F4 knockdown. (J) MTT assays in HEY cells overexpressing E2F4 with or without co‐transfection of SNRPF‐targeting siRNA. (K‐L) Representative images of (K) colony formation and (L) Transwell migration/invasion assays in HEY cells treated as described in panel (J). Corresponding statistical quantification is provided in Figure S7E–G. Statistical significance was determined using the two‐tailed Student's t‐test for simple comparisons between two groups (E and F), one‐way ANOVA followed by Dunnett's post‐hoc test (H), and two‐way ANOVA followed by Tukey's post‐hoc test (J). The correlation between gene expression levels in clinical samples was assessed using Pearson correlation analysis (G). ^**^ *p* < 0.01.

To test this hypothesis, we searched for potential E2F4‐binding motifs within the 1500‐bp promoter region of SNRPF using the JASPAR database. This in silico analysis identified multiple putative E2F4‐binding loci (Figure 7A), with the highest‐scoring candidates sharing the core MA0470.1 motif (Figure 7B). Supporting this prediction, E2F4 ChIP‐seq datasets retrieved from the Cistrome Data Browser exhibited distinct E2F4‐binding peaks at the SNRPF promoter across various cell lines, including HeLa‐S3, K562, and MCF‐7 (Figure 7C). To experimentally validate this direct physical interaction in OC, we performed ChIP‐qPCR assays utilizing an endogenous E2F4‐specific antibody in SKOV3 and HEY cells. Based on the JASPAR scores, specific primers were designed targeting the top two predicted regions: Site #1 (GGGCGGGGGTG) and Site #2 (AGGCAGGAAGG) (Figure 7D). The results demonstrated robust enrichment of E2F4 at Site #2 compared to the IgG control, whereas no evident binding was observed at Site #1. This finding indicates that E2F4 selectively occupies the SNRPF promoter solely at Site #2 (Figure 7E).

To determine whether this physical occupancy functionally drives transcriptional activation, we generated a wild‐type (WT) luciferase reporter construct encompassing the SNRPF promoter sequence spanning Site #2. A corresponding mutant (MT) reporter was constructed by deleting this specific binding motif. Dual‐luciferase reporter assays performed in HEK293T cells revealed that targeted silencing of E2F4 markedly hindered the transcriptional activity of the WT promoter (Figure 7F). In contrast, this suppressive effect was substantially blunted in the MT construct (Figure 7F). These functional data mechanistically establish that E2F4 directly activates SNRPF transcription via specific engagement with Site #2.

Clinically, correlation analysis uncovered a strong positive association between E2F4 and SNRPF expression levels in OC patient samples (Pearson r = 0.6730; Figure 7G), further endorsing the relevance of this transcriptional axis. Consistently, siRNA‐mediated E2F4 knockdown significantly reduced both SNRPF mRNA (Figure 7H) and protein levels (Figure 7I) in OC cells. Given that DDX24 sustains E2F4 expression, we further investigated whether the regulation of SNRPF by DDX24 is E2F4‐dependent. Although DDX24 overexpression robustly increased SNRPF protein levels (Figure S7A,B), this effect was virtually abolished upon concurrent siRNA‐mediated knockdown of E2F4 (Figure S7C,D). These data indicate that DDX24 reinforces SNRPF expression through an E2F4‐mediated transcriptional mechanism, thereby establishing a self‐sustaining SNRPF/DDX24/E2F4 positive feedback loop.

To further validate SNRPF as a critical downstream effector of the oncogenic activity of E2F4, we performed functional rescue experiments. The overexpression of E2F4 enhanced malignant phenotypes in HEY cells, including cell proliferation (Figure 7J), colony‐forming capacity (Figure 7K and Figure S7E), and metastatic potential (Figure 7L and Figure S7F,G), whereas cotransfection with SNRPF‐specific siRNA significantly attenuated these effects across all functional assays.

These findings collectively support the conclusion that E2F4 promotes OC progression, at least in part, through direct transcriptional activation of SNRPF.

### 2.10 ASO‐Mediated Knockdown of SNRPF Suppresses OC Progression In Vitro and In Vivo

**FIGURE 8:**
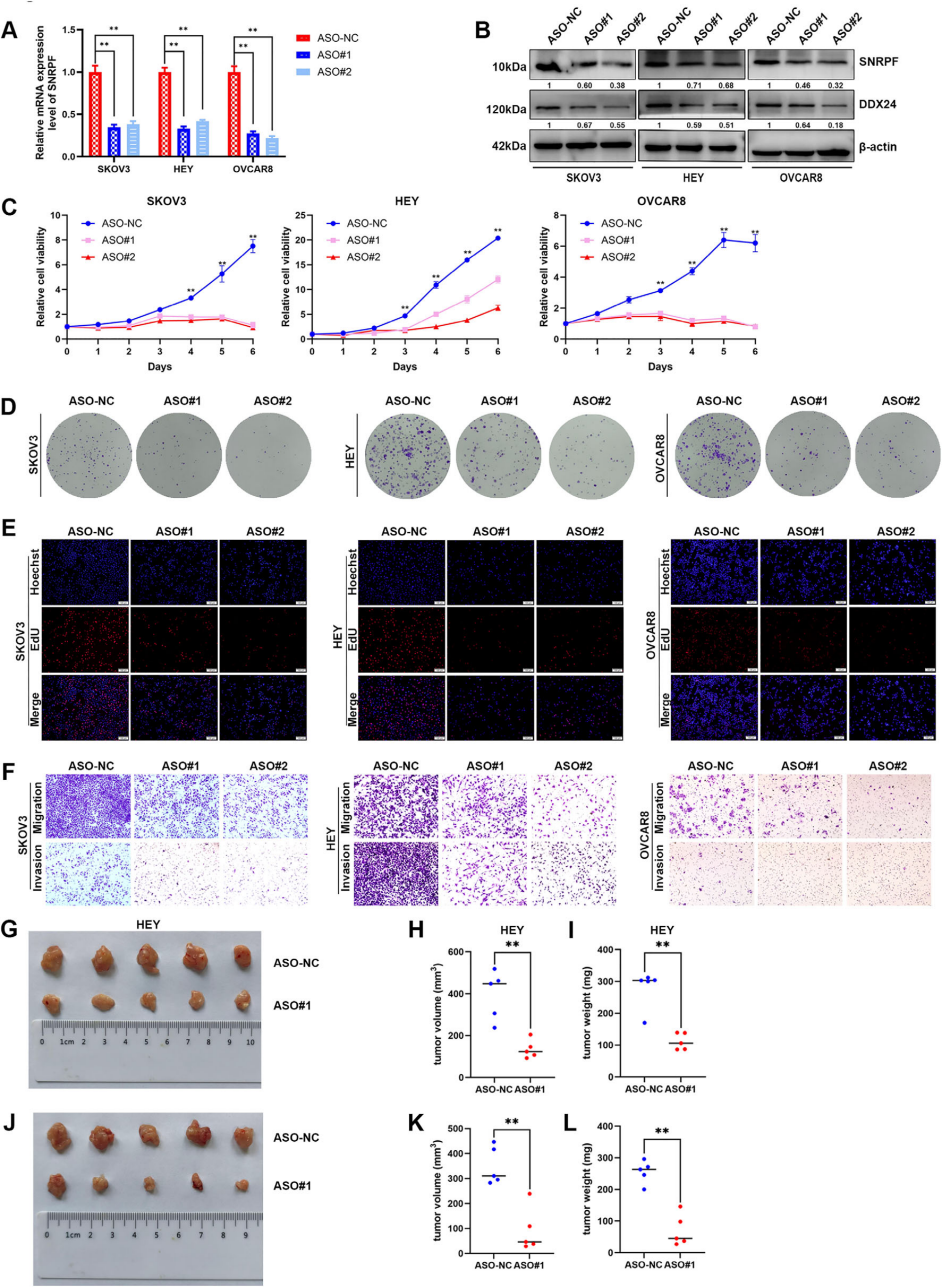
ASO‐mediated Knockdown of SNRPF Suppresses Tumor Growth and Progression of OC Cells In Vitro and In Vivo. (A) qPCR measurement of SNRPF mRNA levels in SKOV3, HEY, and OVCAR8 cells following treatment with SNRPF‐targeting ASOs. (B) Western blotting analysis of SNRPF and DDX24 protein levels in OC cells after ASO treatment. (C) MTT assays evaluating the viability of OC cells treated with ASO‐NC, ASO#1, or ASO#2 over a 6‐day time course. (D) Representative images of colony formation assays assessing the clonogenic capacity of OC cells treated with SNRPF‐ASO or ASO‐NC. Corresponding statistical quantification is provided in Figure S8A. (E) Representative images of EdU incorporation assays indicating DNA synthesis activity in OC cells treated with SNRPF‐ASO. Corresponding statistical quantification is provided in Figure S8B). (F) Representative images of Transwell migration and invasion assays assessing motility and invasive potential in OC cells after SNRPF‐ASO treatment. Corresponding statistical quantification is provided in Figure S8C,D. (G) Representative photographs of excised tumors from the HEY cell‐derived xenograft (CDX) model treated with intratumoral injections of ASO‐NC or SNRPF‐ASO#1 (*n* = 5 per group). (H‐I) Quantification of (H) tumor volume and (I) tumor weight at the endpoint in the HEY CDX model. (J) Representative photographs of excised tumors from the PDX model treated with ASO‐NC or SNRPF‐ASO#1 (*n* = 5 per group). (K‐L) Quantification of (K) tumor volume and (L) tumor weight at the endpoint in the PDX model. Data are presented as the mean ± SD. Statistical significance was determined using two‐way ANOVA followed by Tukey's multiple comparisons test for the growth curves (C), one‐way ANOVA followed by Dunnett's test for (A), and unpaired two‐tailed *t*‐tests for real‐time two‐group in vivo comparisons (H, I, K, L). ^**^ *p* < 0.01.

ASOs represent a promising modality for targeting the transcripts of conventional undruggable proteins and noncoding RNAs. To assess the therapeutic potential of SNRPF silencing, we employed SNRPF‐specific ASOs in OC cell lines SKOV3, HEY, and OVCAR8. qPCR confirmed a significant reduction in SNRPF mRNA levels following ASO treatment (Figure 8A), accompanied by decreased SNRPF protein abundance as detected by Western blotting (Figure 8B). In line with our proposed pathway, SNRPF‐ASO treatment also reduced DDX24 protein levels (Figure 8B).

Functionally, SNRPF silencing markedly inhibited cell proliferation (Figure 8C), colony‐forming capacity (Figure 8D and Figure S8A), DNA synthesis (Figure 8E and Figure S8B), and migration/invasion (Figure 8F and Figure S8C,D). To evaluate antitumor efficacy in vivo, a HEY cell‐derived xenograft (CDX) model was established in immunodeficient NCG mice. Intratumoral administration of SNRPF‐ASO, compared to a nontargeting control ASO (ASO‑NC), significantly suppressed tumor growth (Figure 8G), as evidenced by reduced tumor volume (Figure 8H) and weight (Figure 8I). A similar inhibitory effect was observed in a patient‐derived xenograft (PDX) model (Figure 8J), with decreased tumor size (Figure 8K) and weight (Figure 8L) after SNRPF‐ASO treatment.

These in vitro and in vivo findings demonstrate that ASO‐mediated SNRPF knockdown effectively impairs OC cell proliferation and metastatic potential, supporting SNRPF as a promising therapeutic target in OC.

## 3 Discussion

Dysregulated RNA splicing represents a critical posttranscriptional mechanism that profoundly affects tumor progression and patient prognosis [22]. Among aberrant splicing events, IR has emerged as a conserved and mechanistically important process in cancer. Across eukaryotes, IR can destabilize mRNAs via NMD triggered by PTCs within retained introns, or through nuclear retention and degradation [7, 23]. Aberrant IR patterns have been widely documented in cancers and influence tumor biology by altering tumor‑suppressor activity, oncoprotein production, neoantigen presentation, therapy resistance, and cell proliferation, serving both as diagnostic biomarkers and therapeutic vulnerabilities [18, 19].

In this work, we identify a previously unrecognized oncogenic feedback loop in OC driven by two sequential IR events. SNRPF depletion induces IR in intron 6 of DDX24, which disrupts the integrity of the Helicase_C domain and produces a PTC‑containing, aberrant isoform that is subsequently degraded by NMD. Loss of DDX24, in turn, triggers IR in intron 2 of E2F4, producing another unstable transcript similarly degraded via NMD. Our genetic‐dependency experiments further confirm that SNRPF regulates E2F4 specifically through a DDX24‐dependent mechanism. Importantly, our data reveal that this splicing event operates as a fail‐safe “double‐lock” silencing mechanism: the PTC‐bearing transcripts are primarily eliminated via NMD at the RNA level, and even if “escapee” transcripts undergo forced translation, the resultant truncated proteins are intrinsically unstable and rapidly degraded, thereby serving as a complete functional off‐switch. Because E2F4 directly activates the SNRPF promoter and serves as the indispensable downstream mediator for DDX24, these events form a self‑reinforcing, self‐sustaining “SNRPF–DDX24–E2F4” axis that sustains aberrant splicing programs and malignant phenotypes.

Previous work has identified several spliceosome components as oncogenic drivers—USP39, which promotes tumor proliferation through HMGA2 splicing [24]; BUD31, which regulates exon inclusion in BCL2L12 to resist apoptosis [25]; PQBP1, which induces exon 2 skipping in BAX leading to apoptosis resistance [26]; and SNRPB, which facilitates malignant growth and cisplatin resistance via exon skipping of POLA1 and BRCA2 and modulates IR in DDX39A, affecting ITGA6A expression [27, 28]. The SF3B complex is another critical splicing factor targeted by natural products such as pladienolide B, which enhances immune infiltration and sensitizes tumors to PD‑L1 blockade [29]. Adding to this landscape, our study establishes SNRPF as a pivotal oncogenic splicing factor in OC. Notably, while other Sm family members (e.g., SNRPB, SNRPD1, and SNRPG) are also elevated in OC, our data indicate that only SNRPF consistently governs the DDX24/E2F4 splicing program. This suggests an intriguing degree of substrate preference or a unique regulatory role for SNRPF within the OC‐associated spliceosome, a phenomenon that warrants further structural investigation.

The clinical consequence of this axis is substantial. Our TMA cohort analysis demonstrates that SNRPF overexpression is closely linked to advanced FIGO stages and elevated CA‐125 levels. Although its significance in the multivariable model is attenuated by strong collinearity with these dominant clinical parameters, the univariable results highlight SNRPF as a reliable indicator of poor prognosis. These findings suggest that the SNRPF/DDX24/E2F4 axis may contribute to the acquisition of aggressive clinicopathological features, thereby reflecting the overall disease severity in OC patients. Given the expanding clinical translation of ASO therapies [30], this pathogenic circuit presents a previously elusive therapeutic vulnerability. By demonstrating that SNRPF‐targeted ASOs can dismantle this network and suppress tumor growth across in vitro, CDX, and PDX models, we provide a definitive pharmacological proof‐of‐concept using intratumoral administration. However, we acknowledge that clinical management of OC typically requires systemic administration to address intraperitoneal dissemination. Future efforts will focus on optimizing the delivery of these ASOs—using advanced vehicles such as lipid nanoparticles (LNPs) or specific chemical modifications—to ensure stable systemic distribution and effective tissue penetration in clinical settings.

Mechanistically, DDX24 is a DEAD‑box RNA helicase implicated in multiple aspects of RNA metabolism [20], and contains conserved ATPase/RecA‑like and Helicase_C domains essential for nucleic acid remodeling [21, 31, 32]. Indeed, our functional evaluation of the truncated DDX24‐S isoform corroborated that the structural integrity of the Helicase_C domain is absolutely indispensable for DDX24‐mediated tumor progression, as this PTC‐induced truncation essentially abolishes its oncogenic capacity. While DDX24 has been implicated in LAMB1 mRNA stabilization in hepatocellular carcinoma [20] and in autophagy regulation in lung cancer via IKBKG isoform switching  [33], our findings identify DDX24 as a critical downstream effector of SNRPF in OC, where precise splicing is required for malignant proliferation and invasion.

E2F4, traditionally classified as a transcriptional repressor, is increasingly recognized for oncogenic activities, such as promoting proliferation via DSCC1 in gastric cancer [34] and activating MNX1 in colorectal cancer [35]. Here, E2F4 serves a dual role: its pre‐mRNA operates as a direct splicing target of DDX24, whereas the E2F4 protein acts as a transcriptional activator of SNRPF. Mechanistically, we demonstrated that E2F4 directly occupies specific motifs within the SNRPF promoter to drive its transcription. Importantly, epistatic analyses revealed that the reciprocal regulation of SNRPF by DDX24 is strictly E2F4‐dependent; the DDX24‐induced up‐regulation of SNRPF expression was largely abolished upon concurrent E2F4 depletion. By establishing E2F4 as the requisite downstream mediator for DDX24, these data definitively complete the feedback loop. Crucially, our bidirectional functional rescue experiments confirm this hierarchical dependency: restoring downstream effectors effectively counteracts the tumor‐suppressive phenotypes induced by upstream gene silencing, just as silencing downstream effectors abrogates the tumor‐promoting effects of upstream overexpression. This self‐reinforcing circuit highlights a highly integrated layer of gene expression control, where transcriptional signaling and alternative splicing are interlocked to maintain the malignant phenotype of OC. Furthermore, our study minimizes the possibility of secondary, indirect artifacts commonly associated with broad spliceosome perturbations. By integrating transcript‐specific splicing evaluations (including a customized DDX24‐minigene reporter and endogenous E2F4 RNA transcript analysis), intracellular RIP, and sequence‐specific RNA pull‐down assays, we provide evidence that the sequential splicing events within this axis are mediated through the direct physical binding of SNRPF and DDX24 to the intronic regions of their respective targets. This sequence recognition highlights the mechanistic precision of this regulatory cascade. While our study focuses on the DDX24/E2F4 axis, it is likely that SNRPF regulates a broader landscape of oncogenic transcripts, a possibility we aim to explore using global CLIP‐seq in future studies.

## 4 Conclusion

In conclusion, we define a spliceosome‑dependent positive feedback loop—SNRPF–DDX24–E2F4—that integrates splicing and transcriptional regulation to sustain OC progression. Sequential IR events in DDX24 and E2F4 produce PTC‑containing transcripts rapidly degraded via NMD, thereby linking perturbed splicing–transcription coupling to malignant phenotypes. ASO‑mediated silencing of SNRPF effectively uncouples this oncogenic circuit, suppressing tumor growth and highlighting the therapeutic promise of targeting spliceosome‑driven regulatory networks in OC.

## 5 Experimental Section

### 5.1 Patient Sample Collection

Fresh‐frozen tissue samples were obtained from Qilu Hospital of Shandong University. This study was conducted in accordance with the Declaration of Helsinki and approved by the Institutional Ethics Committee of Qilu Hospital of Shandong University (No. KYLL‐202412‐050). Written informed consent was obtained from all participating patients.

### 5.2 IHC Staining

Paraffin‐embedded tissue sections were first deparaffinized in xylene and then gradually rehydrated through a series of ethanol washes. Subsequent staining procedures were performed using an immunohistochemistry kit (ZSGB‐BIO, Beijing, China). Antigen retrieval was carried out by heating the sections in Tris‐EDTA buffer at pH 9.0. To block endogenous peroxidase activity, the sections were treated with a hydrogen peroxide solution. The samples were subsequently incubated overnight at 4°C with the appropriate primary antibodies. Following thorough rinsing, the sections were incubated with a biotin‐conjugated secondary antibody and then with a streptavidin‐horseradish peroxidase complex. The immunoreactive signal was visualized by applying freshly prepared diaminobenzidine (DAB) substrate. Staining evaluation was performed using a semiquantitative scoring system, which multiplied the staining intensity by the proportion of positive cells detected. The primary antibodies used included anti‐SNRPF (Abcam, ab154870).

### 5.3 Data Mining From the TCGA and CPTAC Databases

The mRNA expression profiles for OC were retrieved from the TCGA dataset via the GEPIA3 platform [36]. Due to the limited availability of transcript‐level normal tissue controls within the TCGA‐OV RNA‐seq dataset, biological normal controls (including ovary and fallopian tube tissues) were supplemented from the GTEx database. To ensure data comparability and minimize batch effects, we utilized the unified TCGA and GTEx RNA‐seq data, which were uniformly re‐analyzed using the Toil pipeline, downloaded directly from the UCSC Xena repository [37]. Differential expression of SNRPF across four molecular subtypes was analyzed using TCGA Affymetrix U133a microarray data [38]. The protein expression information was accessed through the CPTAC database [39]. Additionally, the CSIOVDB OC database was used to examine the expression levels of SNRPF in both OC and normal tissues and to evaluate its association with the FIGO stage [40].

### 5.4 Cell Culture

The SKOV3 (RRID: CVCL_0532) OC cell line was obtained from the Cell Bank of the Chinese Academy of Sciences, and HEY (RRID: CVCL_0297) cells were generously provided by Dr. Liu's laboratory. OVCAR8 (RRID: CVCL_1629) cells were obtained from the Characterized Cell Line Core Facility at MD Anderson Cancer Center. SKOV3 cells were maintained in McCoy's 5A medium (Macgene, Beijing, China), OVCAR8 cells were cultured in RPMI‐1640 medium (Macgene, Beijing, China), and HEY cells were grown in DMEM (Macgene, Beijing, China). All culture media were supplemented with 10% fetal bovine serum, and the cells were incubated under standard conditions at 37°C in a humidified atmosphere containing 5% CO_2_. All cell lines were authenticated via short tandem repeat (STR) profiling and were tested and confirmed to be free of Mycoplasma contamination prior to experiments.

### 5.5 Cell Transfection, Plasmid Construction, and Lentiviral Infection

#### 5.5.1 siRNA and ASO Transfection

siRNA oligonucleotides targeting SNRPF, DDX24, and E2F4 were synthesized by GenePharma (Shanghai, China). ASOs and siRNAs were transiently transfected into cells using Lipofectamine 2000 (Invitrogen, USA) according to the manufacturer's protocols. Detailed sequences for siRNAs and ASOs are provided in Tables S4.

#### 5.5.2 Plasmid Construction

To generate stable knockdown constructs, shRNA oligonucleotides targeting SNRPF and DDX24 were synthesized and ligated into the pLKO.1‐puro or pLKO.1‐neo vectors. The specific sequences are provided in Table S5.

For overexpression and functional studies, a series of full‐length (L), truncated (S), and mutant plasmids (including pcDNA3.1‐DDX24‐L/S, pcDNA3.1‐E2F4‐L/S, and pLVX‐SNRPF) were synthesized and subcloned by BioSune Biotechnology (Ji'nan, China). Specifically, “L” designates the full‐length isoform, while “S” denotes the truncated isoform. Similarly, the wild‐type (WT) SNRPF promoter region containing the E2F4 binding site and its mutant (MT) lacking this site were synthesized and cloned into the pGL4.26 vector for luciferase reporter assays. All recombinant plasmids were verified by Sanger sequencing. Detailed information on all plasmids is provided in Table S6.

#### 5.5.3 Lentivirus Production and Stable Cell Line Generation

Lentiviral particles were produced in HEK293T cells by co‐transfection of the respective lentiviral constructs with the packaging plasmids psPAX2 and pMD2.G. Culture supernatants containing viruses were harvested 24 and 48 h post‐transfection and used to infect OC cells. Stable cell lines expressing the desired overexpression or knockdown constructs were established via selection with puromycin (or neomycin, as applicable) for at least seven days.

### 5.6 RNA Extraction, qPCR, and Western Blotting

#### 5.6.1 RNA Extraction and qPCR

Total RNA was isolated from tissue samples or cultured cells using TRIzol reagent (Invitrogen, USA) and subsequently reverse transcribed into cDNA. qPCR assays were conducted utilizing a QuantStudio 3 system (Thermo Fisher Scientific, Singapore) to quantify gene expression levels. Relative gene expression levels were calculated using the 2^−ΔΔCt^ method, with GAPDH as the internal control. The oligonucleotide sequences, including those for qPCR, RT‐PCR, RIP‐PCR, and ChIP‐qPCR, are comprehensively listed in Table S7.

#### 5.6.2 Western Blotting

Total protein was extracted using RIPA lysis buffer (Beyotime, China) supplemented with protease and phosphatase inhibitors (Beyotime, China). Protein expression was analyzed by Western blotting according to established protocols [27]. Briefly, equal amounts of protein were separated by SDS‐PAGE and transferred onto PVDF membranes (Millipore, Germany). The membranes were blocked with 5% non‐fat milk and incubated overnight at 4°C with the following primary antibodies: anti‐SNRPF (Abcam, ab154870; 1:1000), anti‐DDX24 (Proteintech, 15769‐1‐AP; 1:1000), anti‐E2F4 (Abways, CY8718; 1:1000), and anti‐β‐actin (Sigma–Aldrich, A5441; 1:5000), which served as the loading control. After incubation with HRP‐conjugated secondary antibodies, the protein bands were visualized using an enhanced chemiluminescence (ECL, Millipore, Germany) detection system. For the quantitative analysis of protein expression, densitometric quantification of the Western blotting bands was performed using ImageJ 1.52a software (NIH, USA). The relative protein expression levels were normalized to the band intensity of the corresponding loading control (β‐actin), and the calculated relative ratios are indicated directly below the corresponding bands in the figures. A list of the antibodies used in this study is provided in Table S8.

### 5.7 Assessment of Cell Proliferation and Metastasis

Cell proliferation and metastatic potential were evaluated using MTT (Beyotime, China), colony formation, and Transwell (Corning, USA) assays. The detailed experimental procedures for these assays were based on previously established methods [28]. Briefly, the MTT assay was used to measure cell viability through the metabolic reduction of MTT reagent, the colony formation assay was used to assess the ability of single cells to grow into colonies over time, and the Transwell assay was used to quantify cell migration or invasion through membrane pores in response to chemoattractant stimuli.

To quantify the colony formation ability, the number of visible colonies (defined as clusters containing ≥ 50 cells) was manually counted. The experiments were performed in triplicate, and the average number of colonies was calculated for statistical analysis. For the statistical quantification of cell migration and invasion, cells that successfully migrated or invaded to the lower surface of the porous membrane were imaged under a microscope. The number of cells was counted in at least three randomly selected fields per chamber, and the average cell counts were used for subsequent statistical analysis.

### 5.8 Xenograft Tumor Formation in Nude Mice

Four‐week‐old female BALB/c nude mice were procured from GemPharmatech (Jiangsu, China) for in vivo tumorigenicity studies. HEY cells, previously transfected with either shRNA targeting the gene of interest or control shRNA, were subcutaneously implanted into the axillary region of the mice. After a 15‐day incubation period, the animals were euthanized following ethical guidelines, and the resulting tumors were excised for subsequent measurement and photographic documentation. The Nude mouse xenograft assay was approved by the Shandong University Animal Care and Use Committee (24045). All animal tissues are collected under IACUC protocol.

### 5.9 PDX Model

Fresh tumor specimens from HGSOC patients were cut into small fragments and implanted subcutaneously into immunodeficient NCG mice (GemPharmatech) to establish a PDX model. These xenografts were subsequently expanded via serial transplantation in NCG mice to facilitate ongoing experimental studies. The treatment groups received intratumoral injections of either SNRPF‐ASOs or control ASOs every two days. Upon completion of the treatment regimen, the mice were sacrificed, and the tumors were excised and assessed for both volume and weight to enable downstream analyses. The PDX model assay was approved by the Shandong University Animal Care and Use Committee (24045). All animal tissues are collected under IACUC protocol.

### 5.10 RNA‐seq and Data Analysis

Cells were transfected with siRNAs targeting SNRPF or DDX24 for 48 h, after which total RNA was extracted and sent to BioSune Biotechnology Company for RNA‐seq. Differential expression analysis was conducted using the DEGseq software package. Furthermore, AS events showing significant differences between groups were identified through analysis with the rMATS tool.

### 5.11 Differential Expression Analysis and Candidate Selection

To systematically identify downstream targets, a tiered filtering strategy was applied across different analytical stages:

1. Global Profiling: A high‐stringency threshold of |log_2_FC| ≥ 1.0 (padj < 0.05) was utilized for initial screening in GEPIA3/TCGA datasets and global RNA‐seq visualization (e.g., heatmaps and GO enrichments) to identify predominantly dysregulated genes.
2. Mechanistic Intersection: For the intersection of DEGs with alternative splicing events, an adjusted threshold of log_2_FC ≤ ‐0.58 (corresponding to a 1.5 fold decrease) was applied. This parameter was utilized to account for the more moderate expression shifts typically driven by splicing‐coupled transcriptional regulation, thereby preventing the exclusion of biologically relevant targets.
3. Target Prioritization and Validation: To narrow down the resulting candidate pool for downstream mechanistic investigation, rigorous bioinformatic assessment—including manual evaluation of splicing events via IGV Sashimi plots and the reapplication of the strict expression filter (log_2_FC ≤ ‐1.0)—was conducted. Finally, the prioritized candidates (e.g., DDX24, E2F4) were subjected to orthogonal verification via qPCR across multiple OC cell lines (SKOV3, HEY, and OVCAR8) to confirm experimental consistency.

### 5.12 RNA Alternative Splicing Analysis

To investigate the regulation of AS, a minigene reporter plasmid was constructed within the pcDNA3.1 vector. The DDX24‐minigene comprised a genomic fragment encompassing partial exon 5, exon 6, specifically truncated intron 6 (retaining ∼500 bp of the flanking sequence at each splice site to preserve cis‐regulatory elements), and exon 7. This construct was synthesized (BioSune Biotechnology, China) and sequence‐verified. Detailed plasmid architecture is provided in Table S6. For the splicing assay, pcDNA3.1‐DDX24‐minigene was co‐transfected with either si‐NC or si‐SNRPF into HEY cells. Total RNA was extracted 48 h post‐transfection for cDNA synthesis. Semi‐quantitative RT‐PCR was performed using a forward primer spanning the exon 5‐exon 6 junction and a reverse primer located on exon 7 to amplify the retained (DDX24 IR+, 985 bp) and spliced (DDX24 IR‐, 185 bp) isoforms of the exogenous reporter. Products were resolved on 2% agarose gels. Primer sequences are listed in Table S7.

To analyze the splicing patterns of endogenous genes, total RNA was isolated from treated cells using TRIzol reagent (Invitrogen). Following reverse transcription into cDNA, semi‐quantitative RT‐PCR was conducted using target‐specific primers designed in flanking exons (exon 2 and exon 3) to amplify the AS region encompassing E2F4 intron 2 (sequences provided in Table S7). The PCR products, representing the retained (255 bp) and spliced (117 bp) E2F4 transcripts, were resolved on 2% agarose gels. GAPDH (87 bp) served as the internal loading control. Band intensities were quantified using ImageJ software to determine the relative ratio of E2F4‐S (retained) to E2F4‐L (spliced) isoforms.

### 5.13 RIP Assay

RIP assays were conducted utilizing an EZ‐Magna RIP kit (Millipore, Germany) according to the manufacturer's protocol. Briefly, cells were lysed in complete RIP lysis buffer, and the lysates were incubated overnight at 4°C with 5 µg of either anti‐Flag monoclonal antibody (Sigma‐Aldrich, F1804; Mouse) or a corresponding normal Mouse IgG (as a negative control). The RNA–protein–antibody complexes were captured using Protein A/G magnetic beads. The co‐immunoprecipitated RNA was subsequently extracted via phenol‐chloroform purification and reverse‐transcribed into cDNA. The enrichment of specific RNA targets was quantified by qPCR. The sequences of the RIP‐qPCR primers are listed in Table S7.

### 5.14 RNA Pull‐Down Assay

Biotin‐labeled RNA probes spanning the exon 6–intron 6–exon 7 junctions (including the essential 5' and 3' splice sites) of DDX24 pre‐mRNA, and the exon 2–intron 2–exon 3 junctions of E2F4 pre‐mRNA, were generated via in vitro transcription using the RNA MAX‐T7 kit (RiboBio, Guangzhou, China) according to the manufacturer's instructions. Corresponding antisense RNAs or scrambled non‐target RNA fragments were synthesized and used as negative controls. The biotinylated RNAs were then incubated with extracted nuclear lysates from SKOV3 cells. The RNA–protein complexes were captured using streptavidin‐coupled magnetic beads supplied in the Magnetic RNA‐Protein Pull‐Down Kit (Geneseed, Guangzhou, China). After washing to remove non‐specific binding, the eluted proteins were analyzed by Western blotting using specific antibodies against SNRPF and DDX24, as described in Section 5.6.2.

### 5.15 ChIP‐qPCR Assay

ChIP was performed using the PureBinding Chromatin Immunoprecipitation Kit (Geneseed Biotech, Guangzhou, China) strictly according to the manufacturer's instructions. Briefly, SKOV3 and HEY cells were cross‐linked with 37% formaldehyde and sonicated to shear chromatin into appropriately sized fragments. The fragmented chromatin was then immunoprecipitated using a ChIP‐grade anti‐E2F4 antibody (Cell Signaling Technology, #40291) or normal IgG (as a negative control). The enriched DNA sequences were purified and subsequently analyzed by qPCR. The specific primers used for amplifying the SNRPF promoter regions are in Table S7. The enrichment results were normalized to the input DNA.

### 5.16 Luciferase Reporter Assay

Cells were co‐transfected with the pGL4.26‐SNRPF‐WT or pGL4.26‐SNRPF‐MT reporter plasmids, along with E2F4 siRNAs or their respective negative controls, using Lipofectamine 2000 (Invitrogen). To normalize for transfection efficiency, the pRL‐TK Renilla luciferase plasmid (Promega, Madison, WI, USA) was co‐transfected into each well. 48 h post‐transfection, luciferase activities were determined using the Dual‐Luciferase Reporter Assay System (Promega) according to the manufacturer's instructions. Relative promoter activity was calculated by normalizing the Firefly luciferase signal to the Renilla luciferase signal.

### 5.17 Statistical Analysis

Quantitative data are presented as the mean ± standard deviation (SD). Comparisons between two independent groups were performed using the unpaired two‐tailed Student's t‐test. For comparisons among three or more groups, one‐way ANOVA (for single‐factor comparisons) or two‐way ANOVA (for growth curves) was performed, followed by Tukey's or Dunnett's post‐hoc test for multiple comparisons correction where appropriate. Survival curves were estimated using the Kaplan–Meier method and compared via the log‐rank test. All statistical analyses were performed using GraphPad Prism 9.0 software. A value of ^*^ *p* < 0.05 was considered statistically significant (^*^ *p* < 0.05, ^**^ *p* < 0.01, ns = not significant).

## Funding

This study was supported by the Shandong Provincial Natural Science Foundation (Grant No. ZR2023MH183).

## Conflicts of Interest

The authors declare no conflicts of interest.

## Supporting information




**Supporting File**: advs75627‐sup‐0001‐SuppMat.docx.

## Data Availability

The data that support the findings of this study are available from the corresponding author upon reasonable request.

## References

[1] G. Caruso , S. J. Weroha , and W. Cliby , “Ovarian Cancer,” Jama 334, no. 14 (2025): 1278–1291, 10.1001/jama.2025.9495. 40690248

[2] R. L. Siegel , T. B. Kratzer , A. N. Giaquinto , H. Sung , and A. Jemal , “Cancer Statistics,” CA: A Cancer Journal for Clinicians 75, no. 1 (2025): 10–45, 10.3322/caac.21871. 39817679 PMC11745215

[3] J. J. Turunen , E. H. Niemela , B. Verma , and M. J. Frilander , “The Significant Other: Splicing by the minor Spliceosome,” WIREs RNA 4, no. 1 (2013): 61–76, 10.1002/wrna.1141. 23074130 PMC3584512

[4] C. L. Will and R. Luhrmann , “Spliceosome Structure and Function,” Cold Spring Harbor Perspectives in Biology 3, no. 7 (2011): a003707–a003707, 10.1101/cshperspect.a003707. 21441581 PMC3119917

[5] R. F. Stanley and O. Abdel‐Wahab , “Dysregulation and Therapeutic Targeting of RNA Splicing in Cancer,” Nature Cancer 3, no. 5 (2022): 536–546, 10.1038/s43018-022-00384-z. 35624337 PMC9551392

[6] M. C. Wahl , C. L. Will , and R. Luhrmann , “The Spliceosome: Design Principles of a Dynamic RNP Machine,” Cell 136, no. 4 (2009): 701–718, 10.1016/j.cell.2009.02.009. 19239890

[7] J. J. Wong and U. Schmitz , “Intron Retention: Importance, Challenges, and Opportunities,” Trends in Genetics 38, no. 8 (2022): 789–792, 10.1016/j.tig.2022.03.017. 35466008

[8] P. Huang , F. Wen , N. Tuerhong , Y. Yang , and Q. Li , “Neoantigens in Cancer Immunotherapy: Focusing on Alternative Splicing,” Frontiers in Immunology 15 (2024): 1437774, 10.3389/fimmu.2024.1437774. 39055714 PMC11269099

[9] L. Emilius , F. Bremm , A. K. Binder , N. Schaft , and J. Dorrie , “Tumor Antigens beyond the Human Exome,” International Journal of Molecular Sciences 25, no. 9 (2024): 4673. 38731892 10.3390/ijms25094673 PMC11083240

[10] A. Zhang , K. Miao , H. Sun , and C. X. Deng , “Tumor Heterogeneity Reshapes the Tumor Microenvironment to Influence Drug Resistance,” International Journal of Biological Sciences 18, no. 7 (2022): 3019–3033, 10.7150/ijbs.72534. 35541919 PMC9066118

[11] A. Chari , M. M. Golas , M. Klingenhäger , et al., “An Assembly Chaperone Collaborates with the SMN Complex to Generate Spliceosomal SnRNPs,” Cell 135, no. 3 (2008): 497–509, 10.1016/j.cell.2008.09.020. 18984161

[12] N. Liu , Z. Wu , A. Chen , et al., “SNRPB Promotes the Tumorigenic Potential of NSCLC in Part by Regulating RAB26,” Cell Death & Disease 10, no. 9 (2019): 667, 10.1038/s41419-019-1929-y. 31511502 PMC6739327

[13] Y. Li , Z. Chen , H. Xiao , et al., “Targeting the Splicing Factor SNRPB Inhibits Endometrial Cancer Progression by Retaining the POLD1 Intron,” Experimental & Molecular Medicine 57, no. 2 (2025): 420–435, 10.1038/s12276-025-01407-2. 39910288 PMC11873159

[14] C. Chang , L. Li , L. Su , et al., “Intron Retention of DDX39A Driven by SNRPD2 Is a Crucial Splicing Axis for Oncogenic MYC/Spliceosome Program in Hepatocellular Carcinoma,” Advanced Science 11, no. 35 (2024): 2403387, 10.1002/advs.202403387. 39018261 PMC11425265

[15] F. P. Li , G. H. Liu , X. Q. Zhang , et al., “Overexpressed SNRPB/D1/D3/E/F/G Correlate with Poor Survival and Immune Infiltration in Hepatocellular Carcinoma,” American Journal of Translational Research 14, no. 6 (2022): 4207–4228. 35836882 PMC9274562

[16] G. Liu , F. Li , M. Chen , Y. Luo , Y. Dai , and P. Hou , “SNRPD1/E/F/G Serve as Potential Prognostic Biomarkers in Lung Adenocarcinoma,” Frontiers in Genetics 13 (2022): 813285. 35356432 10.3389/fgene.2022.813285 PMC8959887

[17] Q. Wu , R. Liao , C. Miao , et al., “Oncofetal SNRPE Promotes HCC Tumorigenesis by Regulating the FGFR4 Expression through Alternative Splicing,” British Journal of Cancer 131, no. 1 (2024): 77–89, 10.1038/s41416-024-02689-5. 38796598 PMC11231362

[18] H. Dvinge and R. K. Bradley , “Widespread Intron Retention Diversifies Most Cancer Transcriptomes,” Genome Medicine 7, no. 1 (2015): 45, 10.1186/s13073-015-0168-9. 26113877 PMC4480902

[19] A. C. Smart , C. A. Margolis , H. Pimentel , et al., “Intron Retention Is a Source of Neoepitopes in Cancer,” Nature Biotechnology 36, no. 11 (2018): 1056–1058, 10.1038/nbt.4239. PMC6226333 30114007

[20] T. Liu , H. Gan , S. He , et al., “RNA Helicase DDX24 Stabilizes LAMB1 to Promote Hepatocellular Carcinoma Progression,” Cancer Research 82, no. 17 (2022): 3074–3087, 10.1158/0008-5472.CAN-21-3748. 35763670

[21] J. Gao , B. A. Aksoy , U. Dogrusoz , et al., “Integrative Analysis of Complex Cancer Genomics and Clinical Profiles Using the cBioPortal,” Science Signaling 6, no. 269 (2013): pl1, 10.1126/scisignal.2004088. 23550210 PMC4160307

[22] R. K. Bradley and O. Anczukow , “RNA Splicing Dysregulation and the Hallmarks of Cancer,” Nature Reviews: Cancer 23, no. 3 (2023): 135–155. 36627445 10.1038/s41568-022-00541-7 PMC10132032

[23] H. Zhou and X. W. Deng , “Intron Retention, an Orchestrated Program of Gene Expression Regulation,” BioEssays 47, no. 4 (2025): 202400248, 10.1002/bies.202400248. 39950398

[24] S. Wang , Z. Wang , J. Li , et al., “Splicing Factor USP39 Promotes Ovarian Cancer Malignancy through Maintaining Efficient Splicing of Oncogenic HMGA2,” Cell Death & Disease 12, no. 4 (2021): 294, 10.1038/s41419-021-03581-3. 33731694 PMC7969951

[25] Z. Wang , S. Wang , J. Qin , et al., “Splicing Factor BUD31 Promotes Ovarian Cancer Progression through Sustaining the Expression of Anti‐apoptotic BCL2L12,” Nature Communications 13, no. 1 (2022): 6246, 10.1038/s41467-022-34042-w. PMC9587234 36271053

[26] X. Liu , J. Zhang , Z. Wang , et al., “Splicing Factor PQBP1 Curtails BAX Expression to Promote Ovarian Cancer Progression,” Advanced Science 11, no. 15 (2024): 2306229, 10.1002/advs.202306229. 38342602 PMC11022708

[27] Y. Li , Z. Chen , J. Peng , et al., “The Splicing Factor SNRPB Promotes Ovarian Cancer Progression through Regulating Aberrant Exon Skipping of POLA1 and BRCA2,” Oncogene 42, no. 31 (2023): 2386–2401, 10.1038/s41388-023-02763-x. 37391593

[28] Y. Li , Z. Chen , Y. Gao , et al., “SNRPB‐mediated Regulation of DDX39A Splicing Promotes Ovarian Cancer Progression by Regulating α6 Integrin Subunit Expression,” Oncogene 44, no. 26 (2025): 2170–2185, 10.1038/s41388-025-03386-0. 40216968

[29] S. Wang , Y. Liu , H. Xiao , et al., “Inhibition of SF3B1 Improves the Immune Microenvironment through Pyroptosis and Synergizes with αPDL1 in Ovarian Cancer,” Cell Death & Disease 14, no. 11 (2023): 775, 10.1038/s41419-023-06301-1. 38012150 PMC10682409

[30] X. Lv , X. Sun , Y. Gao , et al., “Targeting RNA Splicing Modulation: New Perspectives for Anticancer Strategy?,” Journal of Experimental & Clinical Cancer Research 44, no. 1 (2025): 32, 10.1186/s13046-025-03279-w. 39885614 PMC11781073

[31] D. Shi , C. Dai , J. Qin , and W. Gu , “Negative Regulation of the p300‐p53 Interplay by DDX24,” Oncogene 35, no. 4 (2016): 528–536, 10.1038/onc.2015.77. 25867071 PMC4603993

[32] F. Bleichert and S. J. Baserga , “The Long Unwinding Road of RNA Helicases,” Molecular Cell 27, no. 3 (2007): 339–352, 10.1016/j.molcel.2007.07.014. 17679086

[33] S. Sun , X. Jing , G. Tong , et al., “Loss of DDX24 Inhibits Lung Cancer Progression by Stimulating IKBKG Splicing‐mediated Autophagy,” Theranostics 15, no. 5 (2025): 1879–1895, 10.7150/thno.102425. 39897555 PMC11780526

[34] S. Baral , Y. Yu , Q. Sun , et al., “Transcription Factor E2F4 Promote Proliferation, Migration, and Invasion of Gastric Cancer Cells by Transcriptionally Activating DSCC1,” International Journal of Biological Sciences 20, no. 12 (2024): 4978–4998, 10.7150/ijbs.99590. 39309429 PMC11414385

[35] J. K. Li , H. Liu , H. W. Zhang , J. Li , and Z. T. Liang , “A Positive Feedback Loop of E2F4‐Mediated Activation of MNX1 Regulates Tumour Progression in Colorectal Cancer,” Journal of Cancer 14, no. 14 (2023): 2739–2750, 10.7150/jca.86718. 37779874 PMC10539396

[36] Y. J. Kang , L. Pan , Y. Liu , Z. Rong , J. Liu , and F. Liu , “GEPIA3: Enhanced Drug Sensitivity and Interaction Network Analysis for Cancer Research,” Nucleic Acids Research 53 (2025): W283–W290, 10.1093/nar/gkaf423. 40396370 PMC12230660

[37] M. J. Goldman , B. Craft , M. Hastie , et al., “Visualizing and Interpreting Cancer Genomics Data via the Xena Platform,” Nature Biotechnology 38, no. 6 (2020): 675–678, 10.1038/s41587-020-0546-8. PMC7386072 32444850

[38] Cancer Genome Atlas Research Network , “Integrated Genomic Analyses of Ovarian Carcinoma,” Nature 2011. 474(7353): 609–615. 21720365 10.1038/nature10166 PMC3163504

[39] J. R. Whiteaker , G. N. Halusa , A. N. Hoofnagle , et al., “Repository of Targeted Proteomic Assays,” Nature Methods 11, no. 7 (2014): 703–704, 10.1038/nmeth.3002. 24972168 PMC4113142

[40] T. Z. Tan , H. Yang , J. Ye , et al., “Microarray Gene Expression Database of Epithelial Ovarian Cancer Subtype,” Oncotarget 6, no. 41 (2015): 43843–43852, 10.18632/oncotarget.5983. 26549805 PMC4791271

