# Supplementary material for: Disruption of the SNRPF–DDX24–E2F4 Feedback Loop Uncouples Splicing and Transcriptional Regulation to Suppress Ovarian Cancer Progression
Source: Adv Sci (Weinh). 2026 May 10;13(44):e23374. doi: 10.1002/advs.202523374 (PMC13336117; doi:10.1002/advs.202523374)
Supplement: Supplementary file 1 [file ADVS-13-e23374-s001.docx]

Supporting Information

**Disruption of the SNRPF–DDX24–E2F4 Feedback Loop Uncouples Splicing and Transcriptional Regulation to Suppress Ovarian Cancer Progression**

*Yingwei Li*, Zhongshao Chen, Qianqian Gao, Yuehan Gao, and Ning Yang*

**Supplementary Figures**

**Figure S1.** Expression profile of SNRPF in OC subtypes and functional characterization following SNRPF knockdown. (A) Distribution of SNRPF mRNA expression levels across distinct histological subtypes of EOC (Clear Cell, Endometrioid, Mucinous, Mucinous-LMP, Serous, and Serous-LMP) analyzed using the CSIOVDB dataset. (B) Statistical quantification of colony numbers from the experiments shown in Figure 2D. (C) Quantitative analysis of the percentage of EdU-positive cells for SKOV3, HEY, and OVCAR8 cells shown in Figure 2E. (D-F) qPCR analysis of SNRPF, EIF4G2, FAM133B, FANCI, IFRD2, MPV17, NUP62, TEFM, and TGFBI mRNA levels in SKOV3 (D), HEY (E), and OVCAR8 (F) cells after SNRPF knockdown. Data are presented as the mean ± SD (*n* = 3). Statistical significance for quantitative data in (B-F) was determined using one-way ANOVA followed by Dunnett's multiple comparisons test. **p* < 0.05, ***p* < 0.01; ns, not significant.

**Figure S2**. *In vitro* functional characterization of DDX24 and its phenotypic interdependence with SNRPF in OC cells. (A) qPCR analysis of DDX24 mRNA expression in SKOV3, HEY, and OVCAR8 cells following transient transfection with control (si-NC) or specific siRNAs (si-DDX24#1, si-DDX24#2). (B) MTT cell viability assays assessing proliferation rates in SKOV3 and OVCAR8 cells over a 6-day course following DDX24 knockdown. (C) Representative images of EdU incorporation assays in SKOV3 and OVCAR8 cells after DDX24 silencing. (D) Quantitative analysis of the percentage of EdU-positive cells from (C) and Figure 3O. (E) Representative images of colony formation assays in SKOV3 and OVCAR8 cells following DDX24 knockdown. (F) Statistical quantification of colony numbers from (E) and Figure 3P. (G) Representative images of Transwell migration assays in SKOV3 and OVCAR8 cells. (H) Statistical quantification of migrated cells from (G) and Figure 3Q (top panel). (I) Representative images of Transwell invasion assays in SKOV3 and OVCAR8 cells. (J) Statistical quantification of invaded cells from (I) and Figure 3Q (bottom panel). (K) Representative images of colony formation assays evaluating the rescue effect of DDX24 knockdown on SNRPF-overexpressing (pLVX-SNRPF) or vector control HEY cells. (L) Statistical quantification of colony numbers from (K). (M) Statistical quantification of migrated cells from Figure 3S (top panel). (N) Statistical quantification of invaded cells from Figure 3S (bottom panel). (O) MTT assay assessing proliferation in HEY cells transfected with si-SNRPF with or without pcDNA3.1-DDX24 restoration. (P) Representative images of colony formation assays evaluating the rescue effects of DDX24 in SNRPF-knockdown HEY cells. (Q) Statistical quantification of colony numbers from (P). (R) Representative images of Transwell migration and invasion assays evaluating the rescue effect of DDX24 restoration in SNRPF-knockdown HEY cells. (S-T) Statistical quantification of migrated (S) and invaded (T) cells from (R). Data represent the mean ± SD (*n* = 3). Statistical significance was determined using two-way ANOVA followed by Tukey’s test (B, O); one-way ANOVA followed by Dunnett’s test (A, D, F, H, and J); and one-way ANOVA followed by Tukey’s multiple comparisons test (L, M, N, Q, S, and T). ***p* < 0.01.

**Figure S3**. Functional characterization of DDX24 isoforms and verification of the DDX24/SNRPF/E2F4 regulatory axis. (A) Western blotting analysis of exogenous DDX24-L and DDX24-S protein expression in HEY, SKOV3, and OVCAR8 cells. Cells were transfected with 3×Flag-tagged pcDNA3.1-DDX24-L or pcDNA3.1-DDX24-S vectors. Note that the DDX24-S expression constructs were engineered to exclude the native sequence downstream of the PTC, and were directly fused to a C-terminal Flag tag to bypass endogenous NMD surveillance. (B) Statistical quantification of colonies from colony formation assays shown in Figure 4R. (C-D) Quantitative analysis of migrated (C) and invaded (D) cells from Transwell assays presented in Figure 4S. (E-F) Western blotting analysis of SNRPF and E2F4 protein levels following SNRPF overexpression in HEY (E) and SKOV3 (F) cells. (G-H) Western blotting analysis evaluating the effect of SNRPF overexpression on E2F4 protein levels under DDX24 knockdown conditions in HEY (G) and SKOV3 (H) cells. The numbers below the bands represent relative protein expression levels quantified by densitometry and normalized to β-actin. Statistical significance was determined using a one-way ANOVA followed by Dunnett’s multiple comparisons test (B, C, and D). **p* < 0.05, ***p* < 0.01; ns, not significant.

**Figure S4**. E2F4 knockdown inhibits the malignant behaviors of OC cells and mediates the oncogenic effects of DDX24. (A) MTT cell viability assays evaluating the proliferation rates of SKOV3, HEY, and OVCAR8 cells transfected with si-NC or si-E2F4 (#1, #2) over a 6-day period. (B-C) Representative images (B) and statistical quantification (C) of colony formation assays in SKOV3, HEY, and OVCAR8 cells following E2F4 knockdown. (D) Quantitative analysis of the percentage of EdU-positive cells from the experiments shown in Figure 5M. (E) Quantitative analysis of migrated cells corresponding to Figure 5N. (F-G) Representative images (F) and corresponding quantitative analysis (G) of Transwell invasion assays in OC cells after E2F4 knockdown. (H) Statistical quantification of colony numbers from the assays shown in Figure 5P. (I-J) Quantitative analysis of invaded (I) and migrated (J) cells from the assays shown in Figure 5Q. Data are presented as the mean ± SD (*n* = 3). Statistical significance was determined using two-way ANOVA followed by Tukey’s multiple comparisons test for (A), one-way ANOVA followed by Dunnett’s test for (C, D, E, and G) , and one-way ANOVA followed by Tukey’s test for (H, I, and J). ***p* < 0.01.

**Figure S5**. E2F4 overexpression rescues the tumor-suppressive effects induced by DDX24 knockdown in HEY cells. (A) MTT assay assessing cell viability in HEY cells transfected with si-DDX24 or control siRNA, with or without pcDNA3.1-E2F4 restoration. (B-C) Representative images (B) and statistical quantification (C) of colony formation assays evaluating the rescue effects of E2F4 in DDX24-knockdown HEY cells. (D-F) Representative images of Transwell migration and invasion assays (D), along with the corresponding quantitative analysis of migrated (E) and invaded (F) cells. Data are presented as the mean ± SD from three independent experiments. Statistical significance was determined using two-way ANOVA followed by Tukey’s multiple comparisons test for (A), and one-way ANOVA followed by Tukey’s multiple comparisons test for (C, E, and F). ***p* < 0.01.

**Figure S6**. Expression of E2F4 isoforms and the evaluation of other Sm ring proteins on the DDX24/E2F4 pathway. (A) Western blotting analysis of exogenous E2F4-L and E2F4-S protein expression in HEY, SKOV3, and OVCAR8 cells. Cells were transfected with 3×Flag-tagged pcDNA3.1-E2F4-L or pcDNA3.1-E2F4-S vectors. Note that the E2F4-S expression constructs were engineered to exclude native downstream PTC contexts to bypass NMD degradation. (B) Statistical quantification of colonies from colony formation assays shown in Figure 6Q. (C-D) Quantitative analysis of migrated (C) and invaded (D) cells from Transwell assays presented in Figure 6R. (E-G) qPCR validation of the knockdown efficiency of SNRPB (E), SNRPD1 (F), and SNRPG (G) using two independent siRNAs in HEY, SKOV3, and OVCAR8 cell lines. All siRNAs achieved significant reduction of target mRNA levels. (H-J) qPCR analysis of DDX24 and E2F4 mRNA expression following the silencing of SNRPB, SNRPD1, and SNRPG in HEY (H), SKOV3 (I), and OVCAR8 (J) cells. Data represent the mean ± SD (*n* = 3). Statistical significance was determined by one-way ANOVA followed by Dunnett’s multiple comparisons test. **p* < 0.05, ***p* < 0.01; ns, not significant.

**Figure S7**. E2F4 mediates DDX24-induced SNRPF upregulation, and SNRPF is indispensable for E2F4-driven malignant phenotypes. (A-B) Western blotting analysis of DDX24 and SNRPF protein levels upon DDX24 overexpression in HEY (A) and SKOV3 (B) cells. Numbers below the blots indicate the relative band intensities normalized to $\beta$-actin.  (C-D) Western blotting analysis showing the effect of DDX24 overexpression on SNRPF protein levels under E2F4 knockdown conditions in HEY (C) and SKOV3 (D) cells. Numbers below the blots indicate the relative band intensities normalized to $\beta$-actin.  (E) Statistical quantification of colonies from colony formation assays shown in Figure 7K. (F-G) Quantitative analysis of migrated (F) and invaded (G) cells from Transwell assays presented in Figure 7L. Data are presented as the mean ± SD from three independent experiments. Statistical significance was determined using one-way ANOVA followed by Tukey’s multiple comparisons test. ***p* < 0.01.

**Figure S8**. Quantitative analysis of the *in vitro* tumor-suppressive effects induced by SNRPF knockdown. (A) Statistical quantification of colony numbers from the colony formation assays shown in Figure 8D. (B) Quantitative analysis of the percentage of EdU-positive cells from the assays shown in Figure 8E. (C-D) Statistical quantification of migrated (C) and invaded (D) cells from the Transwell assays presented in Figure 8F. Data represent the mean ± SD (*n* = 3). Statistical significance was determined using one-way ANOVA followed by Dunnett’s multiple comparisons test. ***p* < 0.01.

**Supplementary Tables**

**Table S1: Correlation between SNRPF expression and clinical pathological characteristics of patients.**

|  | **SNRPF expression (n=105)** | | |
| --- | --- | --- | --- |
| **Characteristic** | **Low expression**  **(n=52)** | **High expression**  **(n=53)** | ***p* value** |
| **Age, year** |  |  | 0.769 |
| ≤55 | 25 (48.1) | 27 (50.9) |  |
| >55 | 27 (51.9) | 26 (49.1) |  |
| **FIGO (2014)** |  |  | **0.023** |
| I and II | 19 (36.5) | 9 (17.0) |  |
| III and IV | 33 (63.5) | 44 (83.0) |  |
| **CA-125, U/mL** |  |  | **0.025** |
| ≤500 | 31 (59.6) | 20 (37.7) |  |
| >500 | 21 (40.4) | 33 (62.3) |  |
| **Tumor size, cm** |  |  | 0.265 |
| ≤8 | 34 (65.4) | 29 (54.7) |  |
| >8 | 18 (34.6) | 24 (45.3) |  |
| **Omental involvement** |  |  | 0.058 |
| No | 24 (46.2) | 15 (28.3) |  |
| Yes | 28 (53.8) | 38 (71.7) |  |
| **Ascites involvement** |  |  | 0.230 |
| No | 15 (28.8) | 10 (18.9) |  |
| Yes | 37 (71.2) | 43 (81.1) |  |

Values are presented as n (%).

FIGO, International Federation of Gynecology and Obstetrics.

*p* values were calculated using Chi-square test.

**Table S2: Univariate and multivariate Cox proportional hazard regression analysis of PFS.**

|  | **Univariate** | | |  | **Multivariate** | |
| --- | --- | --- | --- | --- | --- | --- |
| **Characteristic** | | **HR (95%CI)** | ***p* value** |  | **HR (95%CI)** | ***p* value** |
| **Age, year** | |  | **0.029** |  |  | **0.017** |
| ≤55 | | Reference |  |  | Reference |  |
| >55 | | 1.62 (1.05-2.50) |  |  | 1.75 (1.11-2.78) |  |
| **FIGO (2014)** | |  | **0.001** |  |  | **0.003** |
| I and II | | Reference |  |  | Reference |  |
| III and IV | | 2.95 (1.69-5.14) |  |  | 2.37 (1.34-4.18) |  |
| **CA-125, U/mL** | |  | **0.006** |  |  | **0.011** |
| ≤500 | | Reference |  |  | Reference |  |
| >500 | | 1.86 (1.20-2.89) |  |  | 1.85 (1.16-2.97) |  |
| **Tumor size, cm** | |  | 0.929 |  |  |  |
| ≤8 | | Reference |  |  |  |  |
| >8 | | 1.02 (0.66-1.58) |  |  |  |  |
| **Omental involvement** | |  | **0.001** |  |  |  |
| No | | Reference |  |  |  |  |
| Yes | | 2.32 (1.45-3.70) |  |  |  |  |
| **Ascites involvement** | |  | 0.103 |  |  |  |
| No | | Reference |  |  |  |  |
| Yes | | 1.54 (0.92-2.60) |  |  |  |  |
| **SNRPF expression** | |  | **0.019** |  |  |  |
| Low | | Reference |  |  |  |  |
| High | | 1.68 (1.09-2.59) |  |  |  |  |

PFS, progression‑free survival; HR, hazard ratio; CI, confidence interval;

FIGO, International Federation of Gynecology and Obstetrics.

**Table S3: Univariate and multivariate Cox proportional hazard regression analysis of OS.**

|  | **Univariate** | | |  | **Multivariate** | |
| --- | --- | --- | --- | --- | --- | --- |
| **Characteristic** | | **HR (95%CI)** | ***p* value** |  | **HR (95%CI)** | ***p* value** |
| **Age, year** | |  | 0.059 |  |  |  |
| ≤55 | | Reference |  |  |  |  |
| >55 | | 1.53 (0.99-2.36) |  |  |  |  |
| **FIGO (2014)** | |  | **0.001** |  |  | **0.006** |
| I and II | | Reference |  |  | Reference |  |
| III and IV | | 2.73 (1.57-4.75) |  |  | 2.26 (1.27-4.03) |  |
| **CA-125, U/mL** | |  | **0.010** |  |  |  |
| ≤500 | | Reference |  |  |  |  |
| >500 | | 1.78 (1.15-2.77) |  |  |  |  |
| **Tumor size, cm** | |  | 0.893 |  |  |  |
| ≤8 | | Reference |  |  |  |  |
| >8 | | 1.03 (0.66-1.60) |  |  |  |  |
| **Omental involvement** | |  | **0.001** |  |  | **0.032** |
| No | | Reference |  |  | Reference |  |
| Yes | | 2.19 (1.36-3.52) |  |  | 1.72 (1.05-2.83) |  |
| **Ascites involvement** | |  | 0.203 |  |  |  |
| No | | Reference |  |  |  |  |
| Yes | | 1.40 (0.84-2.33) |  |  |  |  |
| **SNRPF expression** | |  | **0.011** |  |  |  |
| Low | | Reference |  |  |  |  |
| High | | 1.77 (1.14-2.75) |  |  |  |  |

OS, overall survival; HR, hazard ratio; CI, confidence interval;

FIGO, International Federation of Gynecology and Obstetrics.

**Table S4: siRNA and Antisense oligonucleotides sequences used in this study.**

| **siRNA** | **sequence** |
| --- | --- |
| si-SNRPF#1 | 5′- ACAAGGGCUAUCUGGUAUCdTdT-3′ |
| si-SNRPF#2 | 5′- UGUAAUAAUGUCCUUUAUAdTdT-3′ |
| si-DDX24#1 | 5′- UGACAAACUGGACAUCCUUdTdT-3′ |
| si-DDX24#2 | 5′- CACGUACCUCGGAGAUUUAdTdT-3′ |
| si-E2F4#1 | 5′- GAUUUACGACAUUACCAAUdTdT-3′ |
| si-E2F4#2 | 5′- AAGAACUAGACCAGCACAAdTdT-3′ |
| SNRPF-ASO#1 | TAGTCCATTGAGGAAAGGTT |
| SNRPF-ASO#2 | ATTTGCAAGCTGCATGTTCA |

**Table S5: Oligonucleotide sequences for shRNA construction.**

| **Name** | **Sequence (5’-3’)** |
| --- | --- |
| shSNRPF#1-Sense | CCGGGTACAAGGGCTATCTGGTATCCTCGAGGATACCAGATAGCCCTTGTACTTTTTG |
| shSNRPF#1-Antisense | AATTCAAAAAGTACAAGGGCTATCTGGTATCCTCGAGGATACCAGATAGCCCTTGTAC |
| shSNRPF#2-Sense | CCGGTGAACATGCAGCTTGCAAATACTCGAGTATTTGCAAGCTGCATGTTCATTTTTG |
| shSNRPF#2-Antisense | AATTCAAAAATGAACATGCAGCTTGCAAATACTCGAGTATTTGCAAGCTGCATGTTCA |
| shDDX24-Sense | CCGGCGCTCAAGAAAGATGAGGATACTCGAGTATCCTCATCTTTCTTGAGCGTTTTTG |
| shDDX24-Antisense | AATTCAAAAACGCTCAAGAAAGATGAGGATACTCGAGTATCCTCATCTTTCTTGAGCG |

**Table S6: Detailed Information of Synthesized Plasmids**

| Plasmid Name | Vector Backbone | Insert Type | Description/Sequence Information |
| --- | --- | --- | --- |
| pcDNA3.1-DDX24-L | pcDNA3.1 | CDS | Full-length human DDX24 CDS, native stop codon removed for C-terminal Flag tag |
| pcDNA3.1-DDX24-S | pcDNA3.1 | Mutant CDS | Sequence from the start codon to PTC (TAG in intron 6) immediately before the stop codon, fused to C-terminal Flag |
| pcDNA3.1-E2F4-L | pcDNA3.1 | CDS | Full-length human E2F4 CDS, native stop codon removed for C-terminal Flag tag |
| pcDNA3.1-E2F4-S | pcDNA3.1 | Mutant CDS | Sequence from the start codon to PTC (TGA in intron 2) immediately before the stop codon, fused to C-terminal Flag |
| pLVX-SNRPF | pLVX | CDS | Full-length human SNRPF CDS  (RefSeq: NM_003095) |
| pGL4.26-SNRPF-WT | pGL4.26 | Wild-type promoter | SNRPF promoter region containing the wild-type E2F4 binding site |
| pGL4.26-SNRPF-MT | pGL4.26 | Mutant promoter | SNRPF promoter region with the E2F4 binding site deleted |
| pcDNA3.1-DDX24-minigene | pcDNA3.1 | Truncated  pre-mRNA | A genomic fragment spanning partial exon 5 to exon 7, including a truncated intron 6 (with ~500 bp of each flanking sequence) |

**Table S7: Primers for PCR.**

| **Assay** | **Name** | **Sequence (5’-3’)** |
| --- | --- | --- |
| qPCR | SNRPF-qF | GGGAATGGAGTACAAGGGCTATC |
| qPCR | SNRPF-qR | AAAACTTCACCCAGATGTCCAGAC |
| qPCR | DDX24-qF | GTCCTCTGCTTGGACTGGTTCT |
| qPCR | DDX24-qR | GCTGTTTCTGCGTGGACATTCC |
| qPCR | DDX24-IR(+)-qF | GCTTTGAATCCCCAGTCTGC |
| qPCR | DDX24-IR(+)-qR | GTACTTACACTGTGCTACCGG |
| qPCR | DDX24-IR(-)-qF | ATCCATTACCAGGTCCCACG |
| qPCR | DDX24-IR(-)-qR | ATCACATCCTCAGGCCCAAT |
| qPCR | E2F4-qF | CACCACCAAGTTCGTGTCCC |
| qPCR | E2F4-qR | GCGTACAGCTAGGGTGTCA |
| qPCR | E2F4-IR(+)-qF | TGGGCATAGTGGGAGGGTAG |
| qPCR | E2F4-IR(+)-qR | CCTCGATCTCTGCCTTGAGC |
| qPCR | E2F4-IR(-)-qF | CATCCAGTGGAAGGGTGTGG |
| qPCR | E2F4-IR(-)-qR | CTGCAGCTCCTCGATCTCTG |
| qPCR | GAPDH-qF | ACAACTTTGGTATCGTGGAAGG |
| qPCR | GAPDH-qR | GCCATCACGCCACAGTTTC |
| qPCR | NUP62-qF | CCTTCCAACCAGCCACAAGTAC |
| qPCR | NUP62-qR | CGAAGCAAGAGTCGCTGTTCCA |
| qPCR | MPV17-qF | GCAGTTAGCCAACTTCTACCTGG |
| qPCR | MPV17-qR | GCAAGGTGGAAACGATGGAGTG |
| qPCR | FAM133B-qF | CATCAAGCTCTGATTCTTCCAGC |
| qPCR | FAM133B-qR | TCAGTTTCTGACATGGAGCTTTCA |
| qPCR | TGFBI-qF | GGACATGCTCACTATCAACGGG |
| qPCR | TGFBI-qR | CTGTGGACACATCAGACTCTGC |
| qPCR | EIF4G2-qF | CACGCACTCAAACACCACCTCT |
| qPCR | EIF4G2-qR | GGAGTTCTTCCTTTGACGGTGG |
| qPCR | IFRD2-qF | AAGTACCGTGCCAAGGCTGATC |
| qPCR | IFRD2-qR | GTAGAGCACCTCAAAGCCGAAG |
| qPCR | FANCI-qF | GCAAGCTGATGTTCGACTCATGC |
| qPCR | FANCI-qR | AGGCAGCAGATCAGGTTTTGGC |
| qPCR | TEFM-qF | TGAGAAAGCTCCTCAAACCAGAC |
| qPCR | TEFM-qR | CAGTCCAGCACTGTCAACTTACG |
| qPCR | HDAC3-qF | GAGTTCTGCTCGCGTTACACAG |
| qPCR | HDAC3-qR | CGTTGACATAGCAGAAGCCAGAG |
| qPCR | BECN1-qF | CTGGACACTCAGCTCAACGTCA |
| qPCR | BECN1-qR | CTCTAGTGCCAGCTCCTTTAGC |
| RT-PCR | DDX24-RT-F | GTTTGCCCGTCTGGAAGACT |
| RT-PCR | DDX24-RT-R | CAGGCCCAATGAGCATCAGA |
| RT-PCR | E2F4-RT-F | AAGCGGCGGATTTACGACAT |
| RT-PCR | E2F4-RT-R | AATCTCCCGGGTATTGCAGC |
| RT-PCR | GAPDH-RT-F | TGCACCACCAACTGCTTAGC |
| RT-PCR | GAPDH-RT-R | GGCATGGACTGTGGTCATGAG |
| RIP-qPCR | DDX24-RIP-F | GTCCTCTGCTTGGACTGGTTCT |
| RIP-qPCR | DDX24-RIP-R | GCTGTTTCTGCGTGGACATTCC |
| RIP-qPCR | E2F4-RIP-F | CACCACCAAGTTCGTGTCCC |
| RIP-qPCR | E2F4-RIP-R | GCGTACAGCTAGGGTGTCA |
| ChIP-qPCR | SNRPF-qF1 | AAGCAAAAGAAAGGTGGGGC |
| ChIP-qPCR | SNRPF-qR1 | ACGCACTGAAGAGAGTTGGA |
| ChIP-qPCR | SNRPF-qF2 | ACCTCTTTGCGTCCAATCACA |
| ChIP-qPCR | SNRPF-qR2 | CCCGAGCCGCAGTTTCAA |

| **Antibodies used for Western blotting** | | | | |
| --- | --- | --- | --- | --- |
| **Cat.No** | **Antibody** | **Species** | **Dilution** | **Company and description** |
| ab154870 | Anti-SNRPF | Rabbit | 1/1000 | Abcam |
| 15769-1-AP | Anti-DDX24 | Rabbit | 1/1000 | Proteintech |
| CY8718 | Anti-E2F4 | Rabbit | 1/1000 | Abways |
| F1804 | Anti-Flag | Mouse | 1/1000 | Sigma–Aldrich |
| A5441 | Anti-β-actin | Mouse | 1/5000 | Sigma–Aldrich |
| **Antibodies used for IHC, RIP, and ChIP** | | | | |
| **Cat.No** | **Antibody** | **Species** | **Dilution/ amount** | **Company and description** |
| ab154870 | Anti-SNRPF | Rabbit | 1/200 | Abcam, IHC |
| F1804 | Anti-Flag | Mouse | 5μg per reaction | Sigma, RIP |
| #40291 | Anti-E2F4 | Rabbit | 1/50 | Cell Signaling Technology, ChIP |

**Table S8: List of the antibodies used in the study.**
